# Multifunctional PEEK implants via mussel adhesion-mediated assembly for osteoimmune regulation and antibacterial properties

**DOI:** 10.3389/fbioe.2025.1624106

**Published:** 2025-09-09

**Authors:** Lei Wang, Qiang Wang, Fan Wang, Shouliang Xiong, Xin Yang, Jie Zhao, Xiao Lu, Yinchang Zhang, Pingbo Chen, Surong Qian, Guohai Lu, Chengyong Gu

**Affiliations:** ^1^ Department of Orthopedics, The First Affiliated Hospital of Wannan Medical College, Wuhu, Anhui, China; ^2^ Department of Orthopaedics, Shanghai Key Laboratory for Prevention and Treatment of Bone and Joint Diseases, Shanghai Institute of Traumatology and Orthopaedics, Ruijin Hospital, Shanghai Jiao Tong University School of Medicine, Shanghai, China; ^3^ Department of Rehabilitation Medicine, Suzhou Municipal Hospital, Nanjing Medical University Affiliated Suzhou Hospital, Suzhou, Jiangsu, China; ^4^ Orthopedics and Sports Medicine Center, Suzhou Municipal Hospital, Nanjing Medical University Affiliated Suzhou Hospital, Suzhou, Jiangsu, China; ^5^ Anesthesiology Department, Suzhou Municipal Hospital, Nanjing Medical University Affiliated Suzhou Hospital, Suzhou, Jiangsu, China

**Keywords:** immune regulation, implant-associated infections, osseointegration, PEEK, strontium

## Abstract

**Introduction:**

Polyetheretherketone (PEEK) is widely recognized for its exceptional mechanical properties and biocompatibility, making it a promising material for orthopedic implants. However, its inherent biological inertia—characterized by poor osteogenic potential, limited antibacterial activity, and excessive immune activation—compromises its clinical performance.

**Methods:**

To address these limitations, we developed a multifunctional PEEK implant (PEEK-PDA-Sr/AMP) through a mussel-inspired self-assembly process, incorporating strontium ions (Sr^2+^) for dual biological functions and the antimicrobial peptide PMAP-36. A polydopamine (PDA) coating was first applied to enhance microscale surface roughness and hydrophilicity. Subsequently, Sr^2+^ and AMP were immobilized onto the PDA-modified surface.

**Results:**

The resulting PEEK-PDA-Sr/AMP implants significantly promoted the adhesion and spatial organization of bone marrow mesenchymal stem cells (BMSCs) and macrophages (BMMs) in vitro. Furthermore, the modified surface facilitated macrophage polarization toward a pro-regenerative phenotype, thereby fostering an osteoimmune microenvironment conducive to osteogenic differentiation of BMSCs. The functionalized implants also exhibited strong antibacterial efficacy against both *Staphylococcus aureus* and *Escherichia coli*. In a rat model of osteomyelitis, in vivo evaluations via micro-CT, histology, and immunohistochemistry confirmed that the PEEK-PDA-Sr/AMP implants markedly enhanced immunomodulation, bone regeneration, and osseointegration.

**Discussion:**

This study demonstrates a novel surface bioengineering strategy for constructing multifunctional PEEK implants with improved immunomodulatory, osteogenic, and antibacterial properties, offering a promising solution to meet complex clinical requirements in orthopedic applications.

## 1 Introduction

Polyetheretherketone (PEEK) is a high-performance polymer that has been widely employed in orthopedic implants since the late 1990s due to its excellent mechanical strength, chemical stability, biocompatibility, and an elastic modulus comparable to that of natural bone (Zheng et al., 2022; Chen X. et al., 2022). PEEK has demonstrated promising clinical potential in various orthopedic procedures, including fracture fixation, bone defect reconstruction, spinal fusion, and joint replacement, particularly in load-bearing applications where biomechanical compatibility and long-term stability are essential. Compared to conventional metallic or ceramic implant materials, PEEK exhibits a more bone-like elastic modulus, effectively minimizing stress shielding effects and thereby reducing the risks of implant loosening and bone resorption. In addition, PEEK possesses favorable radiolucency (X-ray transparency), which facilitates postoperative follow-up and clinical imaging assessments (Kim et al., 2020; Vanaei et al., 2021). Although it has many advantages above, the widespread clinical use of PEEK is restricted by its hydrophobicity, poor osseointegration and microbial infection susceptibility which pose a significant risk of implant failure (Zheng et al., 2022; Sacks et al., 2024). Giving PEEK implants biological activity to address these drawbacks, including better cell binding, increased osteogenic potential, and even anti-infection properties to thwart the spread of harmful bacteria, have a significant clinical impact on the long-term success of implant surgery (He et al., 2023).

In-depth study has found that immune and inflammatory responses following implantation can obstruct the osteogenesis process, therefore, the concept of osteoimmunology was proposed (Yu et al., 2022; Zhang et al., 2023). When PEEK is implanted, it causes a fibrous capsule to form, triggers the body’s immune system to produce a foreign body reaction (FBR), and prevents PEEK from adhering to bone tissue, which is a multifactorial immune regulation process that involves several cells and cytokines (Zhang et al., 2023). Macrophages are a crucial element of the immune system. They become activated when they participate in inflammatory reactions in local tissue and are categorised as M1 and M2 macrophages according to their polarisation characteristic (Mantovani et al., 2004; Zhao et al., 2024). One factor thought to influence FBR is macrophage polarisation, which is the result of an interplay between M1 and M2 macrophage phenotypes (Zhang et al., 2023). M1 macrophages have a pro-inflammatory phenotype and can produce cytokines, TNF-α, and IL-6, among other harmful reactive oxygen intermediates, which enhancing inflammatory response and suppressing tissue repair (Fontana et al., 2024; Wang T. et al., 2022). On the contrary, M2 macrophages present an anti-inflammatory phenotype and producing bone morphogenetic protein (BMP), VEGF, and TGF-β to reduce inflammation reaction and promoting tissue repair (Zhang et al., 2023; Wang T. et al., 2022). Even though M1 and M2 macrophages are usually thought of as separate phenotypes, alterations brought about by implants may cause both M1 and M2 markers to express simultaneously in the local microenvironment (Zhang et al., 2023; Yang C. et al., 2024). Consequently, “smart” bone repair materials with immunoregulatory effects ought to be able to control macrophages’ transition to the M1/M2 phenotype and promote the regeneration of bone tissue.

To enhance the osseointegration performance of polyetheretherketone (PEEK), various effective strategies have been proposed, including the incorporation of metal ions, pharmaceuticals, bioactive proteins, or functional peptides. These modifications aim to improve the bioactivity of PEEK surfaces and modulate the local immune microenvironment, thereby promoting the polarization of macrophages toward the M2 phenotype, which is associated with tissue repair. Such immunoregulatory effects play a critical role in facilitating bone regeneration and improving the integration of the implant with surrounding osseous tissue (Zhang et al., 2023; Xu et al., 2019; Du et al., 2024). Compared to other bioactive metal ions in repairing bone tissue such as magnesium (Mg), calcium (Ca), silicon (Si), strontium (Sr) getting more and more attention, which may be a more suitable candidate for “immune osteogenesis” for the following reasons: (1) strontium salt (strontium) as a commercial anti-osteoporosis drug is FDA-approved clinical drug for osteoporosis therapy, which has been reported to have modulate bone metabolism effects via promoting osteoblastogenesis and inhibiting osteoclastogenesis (Jacobs et al., 2020; Sun Y. et al., 2021). (2) Sr^2+^ can control the immunological milieu in bone tissue, polarize macrophages towards M2, and upregulate the expression of molecules including vascular endothelial-derived growth factor (VEGF) and bone morphogenetic protein-2 (BMP-2) to facilitate both bone tissue and vascular regeneration, thus forming an osteogenic microenvironment conducive to bone tissue regeneration (Wang et al., 2025; Mo et al., 2023; Wang L. et al., 2023). (3) According to pertinent studies, strontium can block the NF-κB signalling pathway, which lowers the expression of proinflammatory factors including IL-1β and TNF-α, thus reducing population of M1 macrophages and preventing the inflammatory response from progressing (Wang L. et al., 2023; Huang et al., 2020). Overall, strontium can potentially promote bone tissue repair through both ways of direct osteogenesis and immune osteogenesis. Therefore, the two effects of “direct osteogenesis” and “immune osteogenesis” that strontium ions have at the same time can form a synergistic effect on PEEK materials to achieve the best osteogenesis effect.

It has significant clinical implications to preparing bone implants with antimicrobial properties to prevent and treat implant-associated infections (IAI) (Fu et al., 2021). At present, administering high concentrations of antibiotics locally over an extended period during IAI treatment results in both bacterial resistance to antibiotics and cytotoxic damage to bone tissue, which ultimately hinders the healing process of the damaged bone tissue (Shan et al., 2023; Gao and Ma, 2022; Wu et al., 2023). To treat IAI more effectively, it is critical to develop antimicrobial substances with good biocompatibility in replace of antibiotics. AMPs, or antimicrobial peptides, are an element of the biological innate immune system, which shows strong antibacterial activity against a variety of harmful bacteria, biofilms, and prevents bacteria from developing drug resistance (Wu et al., 2023; Lan et al., 2023). The antimicrobial peptide known as porcine cathelicidin PMAP-36, which was initially identified in 1994 and has a proline-induced hinge region and 36 amino acids, is strongly cationic (13^+^), α-helical, and amphipathic (Storici et al., 1994). Based on porcine antimicrobial peptide PMAP-36, monomeric and dimeric forms of PMAP-36 were chemically synthesized. Relative to dimeric forms of PMAP-36, monomeric forms of antimicrobial peptide PMAP-36, which displayed good antibacterial activity and less cytotoxicity (Scocchi et al., 2005). Because of their poor absorption and distribution, which results in limited bioavailability, AMPs are typically unstable in the gastrointestinal tract and other bodily fluids when administered systemically (Costa et al., 2019). Therefore, AMPs were immobilized on the surface of the biomaterials for prevent and treat IAI is a feasible and effective application strategy (Wu et al., 2023).

In recent years, mussel adhesion proteins have drawn a lot of interest because of their capacity to rapidly solidify in humid environments and form strong adhesion interfaces on the surfaces of various substrate materials (Lee et al., 2024; Jia et al., 2019). Further study revealed that mussel foot gland cells could secrete a super powerful slime, which mainly consisted of *Mytilus edulis* foot proteins (Mefps), which were rich in levodopa (3, 4-dihydroxy-L-phenylalanine, L-DOPA). Catechol in the structure of L-DOPA can be oxidized to form quinone, which in turn causes its own cross-linking, and these components can explain the observed adhesion properties (Lin et al., 2007). Dopamine has become a new coating material because of its molecular structure like 3, 4-dihydroxy-phenylalanine. Dopamine forms a polydopamine (PDA) coating through oxidative self-polymerization under weakly alkaline conditions. Currently, the PDA biomimetic coating has been successfully used to modify the surface of biological materials (Jia et al., 2019). Almost any kind of inorganic or organic material can have the PDA coating applied to its surface because of its long-lasting stability and controlled coating thickness (Lee et al., 2024). At the same time, The PDA coating can be used to chelating metal ions and covalently bind biomolecular without the need for extra catalysts or hazardous reagents because of its unique chemical structure and exceptional biocompatibility. It contains numerous functional groups, including as catechols, amines, and imines (Wang L. et al., 2023; Jia et al., 2019; Qin et al., 2021). In addition, the evidence supporting PDA coating’s ability to enhance cell adhesion, distribution, proliferation, and differentiation is mounting (Wang L. et al., 2023; Wang et al., 2019). Due to these outstanding properties such as simplicity, mildness and environmental friendliness, PDA biomimetic chemical modification technology has been widely used to regulate the reaction of cells and tissues to biomaterials and has shown good application prospects in the fields of surface coating and molecular imprinting (Jia et al., 2019; Qin et al., 2021; Wang et al., 2019).

**FIGURE 1:**
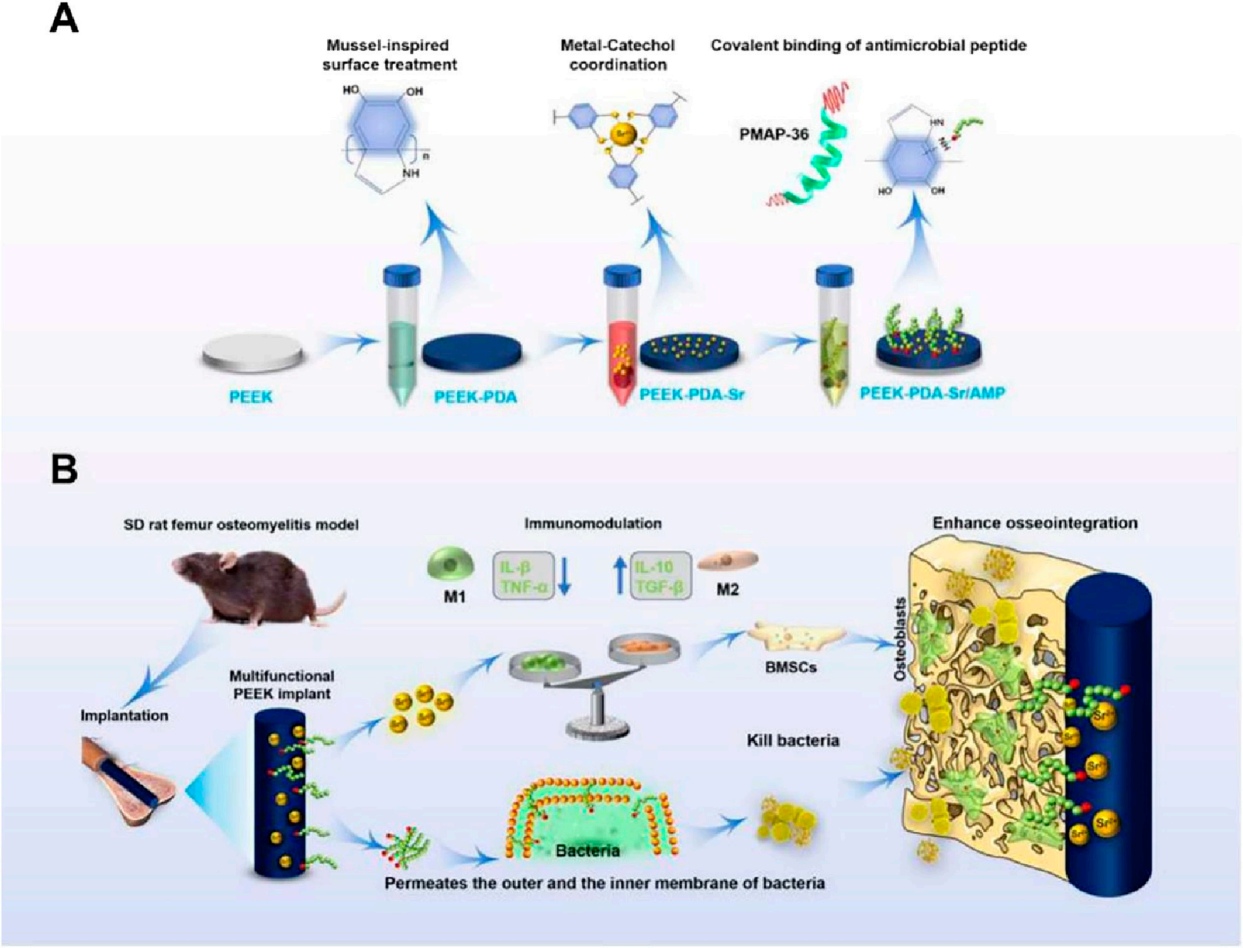
Design strategy of multifunctional PEEK implants. **(A)** Schematic illustration of the mussel-inspired surface modification for Sr ion coordination and chemical graft of antimicrobial peptide on a medical PEEK implant. **(B)** An animal model of implant-related infection, the Sr^2+^ and antimicrobial peptide co-modified PEEK implant shows immunomodulatory, osteogenic, anti-bacterial effects *in vitro* and *in vivo*, synergistically enhancing the osseous integration at the interface between implant and bone tissue after implantation.

In current study, we design multi-functional PEEK orthopedic implants with immunomodulatory, osteogenicity as well as antibacterial properties. The hydrophilic feature of the PEEK surface is achieved through PDA chemical treatment, which is further chelating Sr ion and covalently immobilize monomeric forms of antimicrobial peptide PMAP-36 to fabricate the multifunctional PEEK implants (PEEK-PDA-Sr/AMP). To assess PEEK-PDA-Sr/AMP’s possible immunomodulatory capacity as well as macrophages’ function in osteogenesis, mouse bone marrow macrophages (BMMs) was chosen as immune cells for *in vitro* investigation (Chen Q. et al., 2022). Then, investigations were conducted on the cooperative impact of immunoactive Sr^2+^ on macrophage polarisation and the *in vitro* osteogenesis effect. Next, the antibacterial qualities of the PEEK-PDA-Sr/AMP *in vitro* were further systematically studied. Finally, using a rat femoral implant-related osteomyelitis model, the multifunctionality of the PEEK-PDA-Sr/AMP implant was examined using macrophage polarisation, antibacterial characteristics, and interfacial osteointegration (Figure 1). To the best of our knowledge, this is the first investigation on PEEK implants that have been co-modified with strontium and AMP with multifunction. In addition, this work may offer a potential strategy for surface bioengineering of other inert biomaterials, particularly the need to create multi-functional surfaces for diverse clinical requirements.

## 2 Materials and methods

### 2.1 Materials and equipment

Medical grade PEEK (5.8, 13, and 21.4 mm of diameter and 1 mm of thickness for disks, 1.2 mm diameter and 10 mm length for PEEK rods) were purchased from Junhua High-Performance Specialty Engineering Plastics (PEEK) Products Co. Ltd., Jiangsu, China, and dopamine as well as SrCl_2_ were obtained from Sigma-Aldrich Trading Co. Ltd. Shanghai, China. The antimicrobial peptide PMAP-36 [GRFRRLRKKTRKRLKKIGKVLKWIPPIVGSIPLG-NH_2_], provided by Qiangyao Biotechnology Co., Ltd. (Shanghai, China), was produced with a purity of greater than 95% using a solid phase synthesis technique. In addition, we also purchased α-MEM (Gibco, Grand Island, New York, United States), 4% paraformaldehyde (PFA, Sangon Biotech, United States), fetal bovine serum (FBS, Gibco, United States), 1% penicillin/streptomycin (PS, United States), macrophage-colony stimulating factor (M-CSF, R&D Systems, United States), 2% bovine serum albumin (BSA, Sigma, United States), lipopolysaccharide (LPS, Sigma, United States), FITC-labeled phalloidin (Sigma, United States), 0.2% (v/v) Triton X-100 (Sigma, United States), F4/80 (Abcam, ab6640), CD206 (ab64693, Abcam, United Kingdom), 0.22 µm filter (Millipore, Ireland), iNOS (CST, D6B6S, United States), cell counting kits-8 (CCK-8, Dojindo Laboratories, Tokyo, Japan), CD206 (CST, 24595, United States), 4,6-diamidino-2-phenylindole (DAPI), CD86 (ab220188, Abcam, United Kingdom), phosphate buffer saline (PBS, United States), IScript™cDNA Synthesis Kit (BIO-RAD Technologies), goat anti-mouse IgG (Alex 488, Invitrogen, A11001, United States), SsoAdvanced SYBR Green Supermix (BIORAD Technologies), bovine serum albumin (BSA, Sigma, Shanghai, China), ALP staining kit (Beyotime, China), alizarin red dye (Cyagen, United States), Live/Dead BacLight bacteria viability kit (Invitrogen), BMP-2 (ab214821, Abcam, United Kingdom), goat anti-rabbit IgG (Alex 555, A21434, Invitrogen, United States), *E. coli* (ATCC 25922, United States) and *Staphylococcus aureus* (ATCC 25923, United States).

The research equipments included energy dispersive X-ray analysis (EDX, QX200, Bruker, Germany); scanning electron microscopy (SEM; S-3400, Hitachi, Tokyo, Japan); ICP-AES (Leeman, Ohio, United States); atomic force microscopy (AFM, XE-100, Park Systems, United States); Micro-CT (Bruker Kontich, Belgium); X-ray photoelectron spectroscopy (XPS; PHI 5802, Physical Electronics, London, United Kingdom); laser confocal microscopy (LSCM, Zeiss, Germany); microscope (Axioskop 40 FL, Zeiss); contact angle instrument (Kruss, Germany); video camera (Soft Imaging System); spectrophotometric microplate reader (Bio-Rad 680, CA, United States); and fluorescence microscopy (AMG, Thornwood, NY, United States).

### 2.2 Preparation of PEEK implants

PEEK disks (diameter: 5.8 mm, 13 mm, 21.4 mm, thickness: 1 mm) were utilized for *in vitro* experiments and PEEK rods (diameter: 1.2 mm, length: 10 mm) were utilized *in vivo* investigations. All above PEEK materials were firstly polished and washed sequentially with acetone, ethanol, and deionized water under ultrasonication. To create polydopamine (PDA)-modified PEEK (PEEK-PDA), PEEKs were immersed in dopamine-Tris-HCl buffer solution (2 mg/mL, pH = 8.5) for 24 h in dark environment and were cleaned in an ultrasonic water bath and lastly dried in a vacuum oven. The PEEK-PDA was then incubated for 1 h in a 0.1 M SrCl_2_ solution to produce PEEK-PDA that had been modified with strontium (PEEK-PDA-Sr). For the modification of AMP, PEEK-PDA-Sr disks or rods were incubated with antimicrobial peptide PMAP-36 (2 mg/mL) in PBS solution and the peptide grafting reaction was conducted for 12 h while being shook on a rocker. After removing the free AMP, the discs or rods were labelled PEEK-PDA-Sr/AMP, cleaned three times with distilled water, and dried with N_2_ before being used again. Room temperature was used for all the studies.

### 2.3 The physicochemical characterization of PEEK implants

Using the SEM and AFM, the surface morphology and roughness of PEEK materials were examined. Furthermore, the ions’ distribution on the PEEK implants’ surface was examined using the EDX to verify the existence of the PDA and Sr/AMP coating on the PEEK-PDA-Sr/AMP implant. Using a sputtering rate of 4 nm/min, X-ray photoelectron spectroscopy (XPS) was utilized to analyze the atomic chemical composition of PEEK implants and quantify the concentration of N, O, C, and Sr. Using DI water as the media, a goniometer was utilized to measure the water contact angles of PEEK implants.

### 2.4 Release behavior of Sr and AMP

For the detection of Sr ion and AMP release, the PEEK-PDA-Sr/AMP materials were incubated in 10 mL PBS at 37°C. ICP-AES was utilized to measure the Sr^2+^ concentration and the cumulative percentage of AMP released from the PEEK-PDA-Sr/AMP implants independently. Three parallel measurements were made on days 1, 3, 5, 7, 14, and 21.

### 2.5 Biocompatibility assessment of PEEK implants

Male Sprague-Dawley rats, aged 4 weeks, had their bone marrow removed and were transformed into a suspension of a single cell. Following isolation, the bone marrow stem cells (BMSCs) were cultivated in α-MEM medium supplemented with 1% PS and 10% FBS. Furthermore, 4-week-old mice’s bilateral femurs were isolated. The macrophages generated from bone marrow (BMMs) were cultured in α-MEM medium supplemented with 1% PS, 10% FBS, and 10 ng/mL M-CSF. Both BMMs and BMSCs were grown in 37°C, 5% CO_2_, and 95% humidity incubators.

To investigate the cell morphologies on the various PEEK surfaces, the samples were meticulously rinsed thrice with PBS following a 24-h coculture period in which BMSCs and BMMs were seeded at a density of 3 × 10^4^ in each well. To fix the cells of the experiment, 2.5% glutaraldehyde was then steeped in them for an hour at room temperature. The cells were then dehydrated for 30 min at each concentration in an increasing ethanol gradient (30%, 50%, 70%, 90%, and 100%). Finally, all samples were examined using SEM to analyze cell morphologies after air-drying and gold sputtering.

To observe the filamentous actin of the cytoskeleton of BMSCs, 24 well-plates containing 3 × 10^4^ BMSCs were planted for 24 h on several PEEKs. After that, PBS was utilized thrice to wash the samples. Subsequently, cells in the samples were fixed with 4% paraformaldehyde for 20 min and then washed with PBS. They were permeabilized for 25 min with 0.2% Triton X-100 and then blocked with 0.1% BSA at room temperature under light protection. Afterward, BMSCs were stained with DAPI and treated with FITC-labeled phalloidin. The cytoskeletal arrangement of BMSCs was assessed using an EVOS fluorescence microscope to capture the images.

Both BMSCs and BMMs were cultivated with PEEKs and the cytotoxicity was evaluated using CCK-8 method. Briefly, on these implants, BMSCs and BMMs were planted at a density of 3 × 10^3^ cells/well in a 96-well plate, and they were cultivated for 1, 4, and 7 days. Samples were cleaned with PBS and the medium was replaced with reaction regulator at the predetermined time point for 2 h in the dark according to the instruction. After that, 100 μL of the solution was put into a 96-well plate, and the absorbance at 450 nm was measured using a microplate reader. The cytotoxicity of PEEK materials was then assessed using the subsequent formulation:

$$\text{Relative growth rate}=\text{Optical density }\left(\text{OD}\right)\text{ samples}/\text{Optical density }\left(\text{OD}\right)\text{ blank}\times 100\%$$

where ODsample is the optical density of the PEEK materials, namely, PEEK, PEEK-PDA, PEEK-PDA-Sr and PEEK-PDA-Sr/AMP samples, the optical density of the blank control sample was labeled as ODblank (a-MEM medium).

### 2.6 PEEK implant effects on inflammation response and polarization in BMMs

The expressions of the CD206 (markers of M2) and iNOS (markers of M1) were examined by immunofluorescence analysis. In short, a 24-well plate was used to incubate 3 × 10^4^ cells/well BMMs on PEEK implants. The medium was then supplemented with 100 ng/mL LPS for 8 h to differentiate BMMs into M1 subtypes. The PEEK implants were then treated thrice using PBS. After that, α-MEM medium was added, and the mixture was cultivated for a full day. The BMMs were then treated with 4% PFA for 1 day at 4°C, permeabilized for 5 min with 0.2% (v/v) polyethylene glycol tert-octylphenyl ether and blocked for 1 h with 2% BSA. Primary antibodies (iNOS, F4/80, and CD206) were incubated with the BMMs for a full day at 4°C. Subsequently, they underwent three rounds of washing in phosphate-buffered saline solution before being incubated with secondary antibodies (goat anti-mouse and goat anti-rabbit IgG) for an hour at room temperature. Lastly, DAPI was utilized to stain the BMMs’ nucleus, and the LSCM examined the immunostaining of the BMMs to assess the expression of CD206 and iNOS on macrophages. The fluorescence intensity of various manufacturers was quantified with ImageJ (NIH, version 1.51a) software. To replicate the acute inflammatory response state produced by various PEEK implants, BMMs were grown in a 6-well plate at a density of 1 × 10^5^ cells/well on the PEEK materials. After 2 days of co-cultivation, BMMs were harvested and subjected to TRIzol reagent lysing. Using the UV/VIS, the purity of ribonucleic acid (RNA) was measured. According to the instructions of the reverse transcription polymerase chain reaction (RT-PCR), the primers for IL-10, interleukin-1 beta (IL-1β), transforming growth factor beta (TGF-β), and tumour necrosis factors-alpha (TNF-α), and were displayed in Supplementary Table S1. The ∆∆Ct approach revealed that the expression levels of the housekeeping gene, that is glyceraldehyde 3-phosphate dehydrogenase (GAPDH), to normalize the expression of the interesting gene.

### 2.7 Osteogenesis abilities of PEEK implants in BMSCs

In a 6-well plate, 1 × 10^5^ cells/well BMMs were cultured for 24 h on several PEEK implants. Subsequently, the medium was purified by centrifuging at ×60 g for 5 min. It was then stored at −80°C freezer for future utilization. Prior to BMSC culture, the medium was sterilized with a filter (0.22 μm, also known as macrophage-conditioned media; MCM) and combined with new α-MEM at a 1:2 volume ratio. First, BMSCs at a density of 1 × 10^5^ cells/well were planted on surfaces of different PEEKs materials with α-MEM complete medium and cultured in 6-well plate for 24 h. Subsequently, the osteogenic components (0.1 μM hexahedral 0.25 mM ascorbic acid, and 10 mM 2-(dihydrogen phosphate)-1,2,3-propanetriol) were added to the macrophage-conditioned media prior to its medium replacement. The BMSCs were harvested and lysed after being co-cultured for 7 and 14 days, and then the total RNA was extracted. The primers for the genes runt-related transcription factor 2 (Runx2), osteopontin (OPN), alkalized phosphatase (ALP), osteocalcin (OCN), and collagen-1 (Col-I) were displayed in Supplementary Table S2 after the RT-PCR was conducted in compliance with the guidelines. The target gene’s expression levels were normalized using the housekeeping gene β-actin’s expression levels.

In a 24-well plate, BMSCs were cultivated on the PEEK materials at a density of 5 × 10^4^ cells/well. The BMSCs were then co-cultured for 7 and 14 days before being fixed and processed with ALP or Alizarin Red staining (ARS), respectively. Under a microscope, the expression levels of ALP and ARS were investigated. An ALP assay kit from Beyotime, China, was used to quantify the ALP activity. The BMSCs were treated with 1 w/v% hexadecyl pyridinium chloride following ARS staining. Subsequently, 100 µL of the above supernatant was moved to a fresh 96-well plate, and its absorbance was gauged to determine the extent of ARS expression at 570 nm.

### 2.8 Anti-infection evaluation of various PEEK implants

The antibacterial assays *in vitro* were performed using *S. aureus* and *E. coli*. Totally, PEEK implants were co-cultured for 24 h at 37°C with 1 × 10^5^ CFU/mL of *S. aureus* and *E. coli*, respectively. The samples were then subjected to three PBS cleans, 2.5% glutaraldehyde processing, and graded ethanol dehydration. After applying a gold coating to the PEEK implants, the SEM was used to examine the morphology of the bacteria. Bacteria attached to the various PEEK implants were stained using a live/dead analysis kit following the manufacturer’s instructions and examined with LSCM. To measure the antimicrobial efficacy of different PEEK materials, they were first sterilized and then co-cultured for 24 h with *S. aureus* and *E. coli* (100 μL/cm^2^ and 1 × 10^6^ CFU/mL), respectively. The living bacteria were then extracted from the suspension and collected. Following a further 24 h of culture for the live bacteria, the agar plates were imaged, and the antibacterial rate was computed with the following formula:

$$\text{Antibacterial rate }\left(\%\right)=\left({\text{CFU}}_{\text{Control}}‐{\text{CFU}}_{\text{experimental}\;\text{group}}\right)/{\text{CFU}}_{\text{Control}}\times 100\%$$

CFU_Control_: the colony-forming unit of bacterial on the bare PEEK materials. CFU_experimental group_: the colony-forming unit of bacterial on the modified PEEK materials.

### 2.9 Animal model

All animal experiments were approved by the Institutional Animal Care and Use Committee of Wannan Medical College (Anhui, China) and all experimental procedures followed the NIH guidelines in current paper. Thirty-two male Sprague Dawley rats, all pathogen-free, weighing 300–350 g, were split into four groups at random, with eight rats in each, to create an animal model of implant infection. All rats fasted overnight before surgery. In brief, SD rats were anesthetized and surgery was performed under sterile conditions. Before surgical drape, the hind limbs were shaved and disinfected with povidone iodin. Subsequently, the distal intercondylar fossa of the femur was carefully exposed through longitudinal skin and muscle incisions at the knee flexion position. Then, a bone defect (diameter: 1.2 mm, length: 10 mm) was established using a ring drill parallel to the femoral shaft in the intercondylar fossa followed by 20 μL of *S. aureus* suspension at a concentration of 1 × 10^4^ CFU/mL was injected into the bone defect. Afterward, PEEK materials were inserted into the bone defective area, respectively. Lastly, outer opening of intramedullary cavity was seal by the bone wax and the surgery was sutured with 4–0 nylon. After surgery, the rats were allowed to move around the cage at will without dietary restrictions, and no antibiotics were administered postoperatively.

### 2.10 Micro-CT analysis

The rats were sacrificed at 4 and 8 weeks after the surgery, and femur samples were taken. The harvested femurs were scanned using micro-CT with parameters set to 65 kV, 1 mm Al filter, and 18 μm resolution. Three-dimensional (3D) images were created by reconstructing the two-dimensional (2D) images that were collected. Trabecular separation (Tb.Sp), trabecular thickness (Tb.Th), Bone volume/total volume (BV/TV), andtrabecular number (Tb.N) in the regions of interest (ROI) were utilized to quantitatively evaluate by CTAn software. Each group had at least four parallels.

### 2.11 The effects of PEEK implants on polarization response *in vivo*

Following a 4-week surgical period, rats were slaughtered, and their legs were gathered. For 4 weeks, the specimen was cultured in 10% EDTA. Subsequently, the PEEK implants were extracted with caution so as not to damage the adjacent bone tissues. The specimens were then processed to the section (5 μm thick) using graded ethyl alcohol and paraffin. CD86 (M1 maker) and CD206 (M2 maker) were used to stain the aforementioned sections in order to assess the polarization of macrophages surrounding the PEEK implants. Four sections from different animals were chosen for semi-quantitative analysis, and ImageJ software was utilized to count CD86 and CD206 positive cells in each slice.

### 2.12 Pathological histology and immunohistochemistry

Same as before, the specimen was taken at 4 and 8 weeks following the procedure, and the PEEK implants were carefully removed so as not to harm the surrounding bone tissues. The specimen was subsequently embedded in paraffin and processed into the segment (5 μm thick) after being fixed and decalcified for 4 weeks. Lastly, the sections underwent processing using Hematoxylin-Eosin (H&E), Giemsa, and Masson staining, in that order. To assess bone regeneration, the slices were also immunohistochemically stained with BMP-2. Four sections from different animals were chosen for semi-quantitative examination, and ImageJ software was utilized to count the bone area and BMP-2 positive cells.

### 2.13 Statistical analysis

The data were emerged as mean ± standard deviation (SD). Four biological replicates were used *in vivo* and three biological replicates *in vitro* for the experiments. With SPSS 20 statistical software (version 20.0), the statistical assessment was done by a One-way analysis of variance (ANOVA) with Tukey’s post-test to determine the groups difference. P values inside each Figure panel are indicated by the following notations: **P < 0.01 (moderately significant) and *P < 0.05 (significant).

## 3 Results

### 3.1 The physicochemical characterization of PEEK implants

**FIGURE 2:**
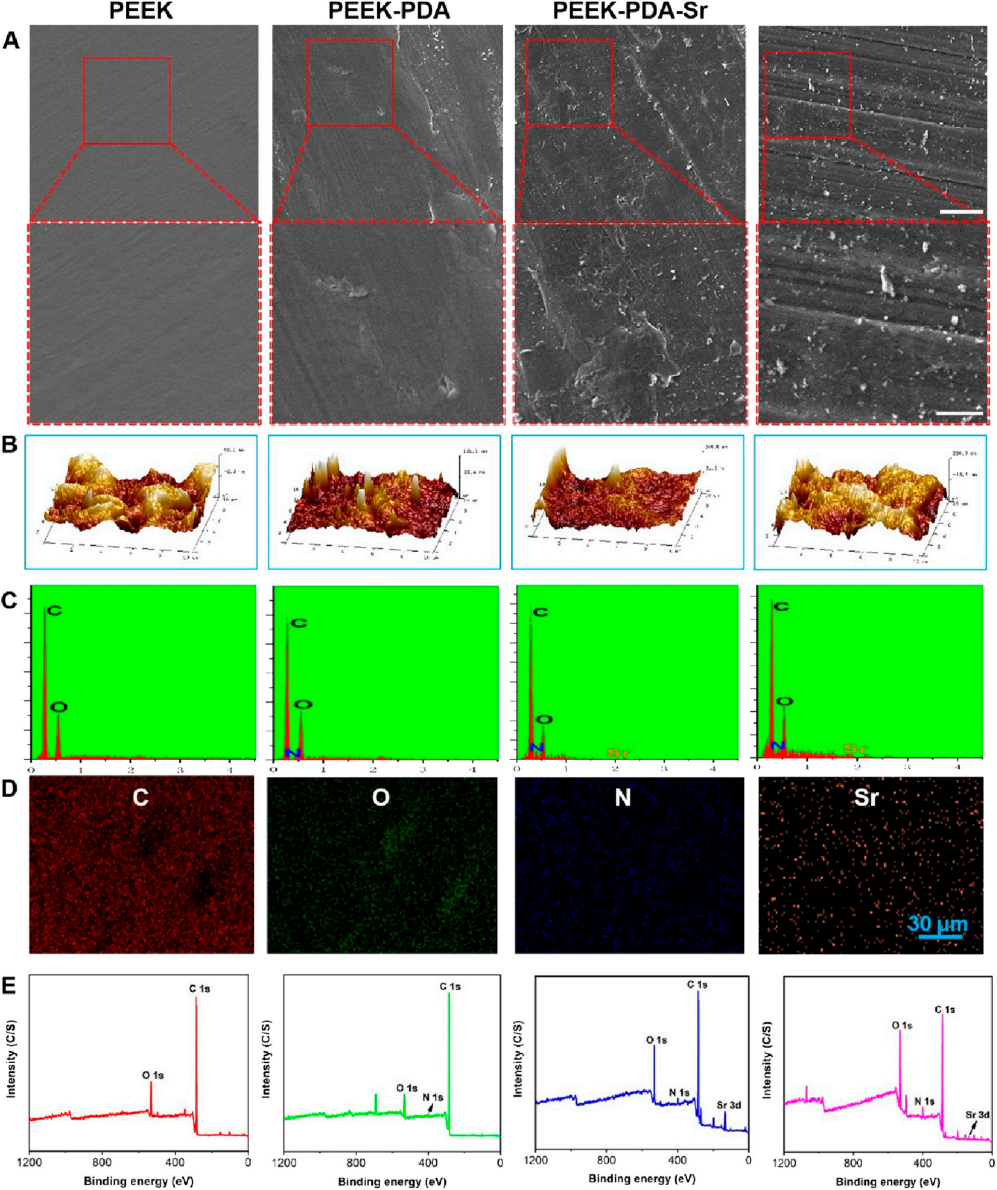
Characterization of various PEEK surfaces. **(A)** SEM image (Scale bars, 30 μm and 10 µm). **(B)** 3D topographical AFM images. **(C)** EDS spectra of different PEEKs. **(D)** SEM-EDS elemental mapping for the Sr^2+^ and AMP co-modified surface (PEEK-PDA-Sr/AMP), scale bar = 30 µm. **(E)** Full XPS spectra of all samples.

**FIGURE 3:**
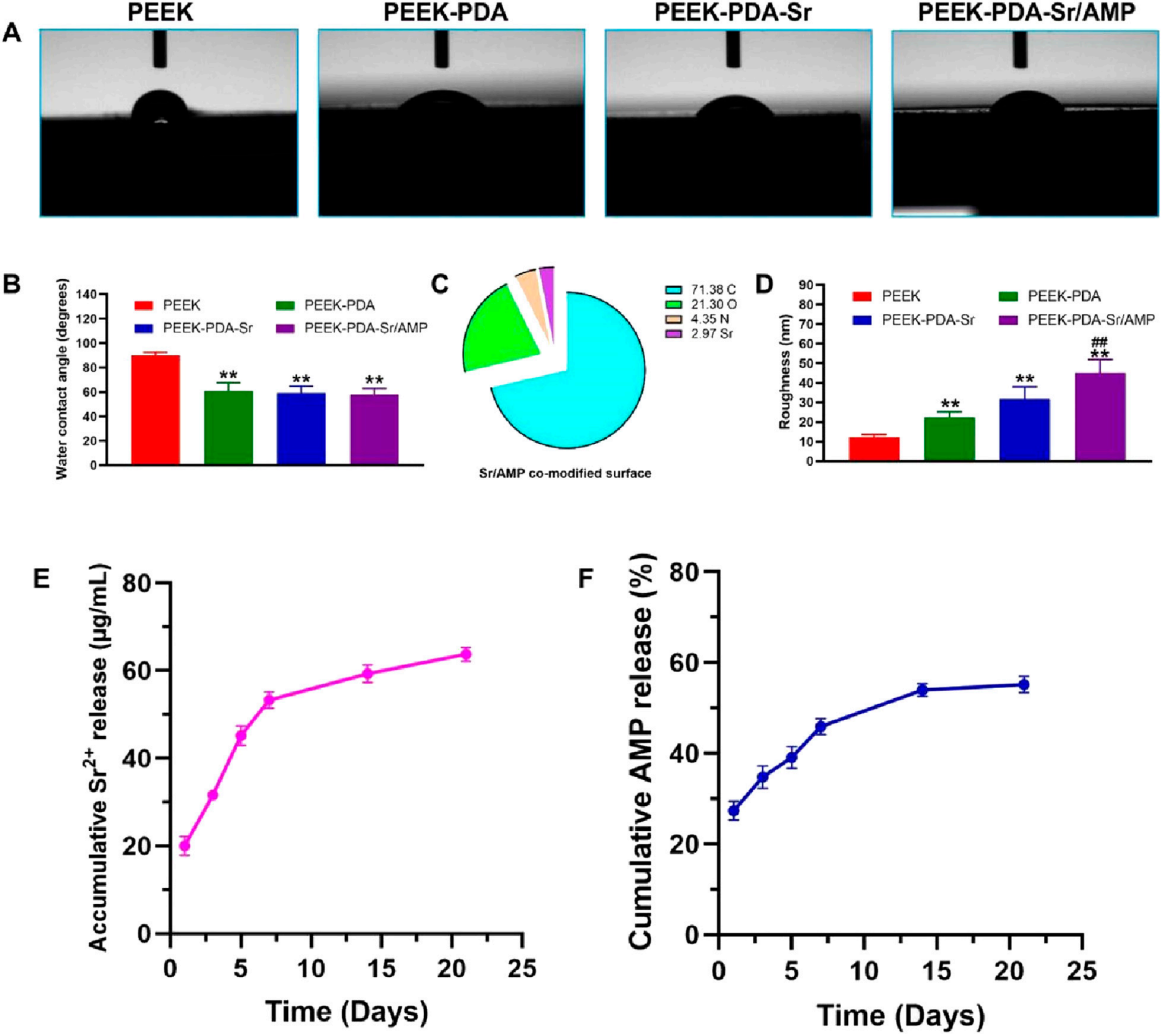
Characterization of different PEEK surfaces. **(A,B)** Water contact angles of the different PEEKs. **p < 0.01 vs. PEEK (*n* = 3). **(C)** XPS quantitative elemental analysis of PEEK-PDA-Sr/AMP. **(D)** AFM quantitative analysis results of the different PEEK surfaces (*n* = 3). **(E)** Cumulative released behavior of Sr from the PEEK-PDA-Sr/AMP. **(F)** Cumulative release behavior of AMP from the PEEK-PDA-Sr/AMP.

AFM and SEM were utilized to examine the surface morphology of PEEK implants both before and after surface treatment. The surface morphology of PEEK implants in the four groups did not differ considerably (Figure 2A). In contrast, PDA-coated PEEK implants displayed darker color compared to bare PEEK groups, which indicates the successful dopamine chemical modification. Using AFM, PEEK implants’ surface micromorphology and roughness were identified (Figure 2B). From Figure 3D, the root means square roughness was 12.2 nm (PEEK) and 22.4 nm (PEEK-PDA), respectively. Following the Sr and AMP treatment, the root means square roughness of implants added up to 31.7 nm (PEEK-PDA-Sr) and 45.1 nm (PEEK-PDA-Sr/AMP). Meanwhile, the EDS verified that PDA and Sr modification were successfully deposited. With the exception of bare PEEK, the nitrogen element can be seen in all PEEK materials with PDA treatment, demonstrating the effectiveness of the PDA coating. Furthermore, the PEEK-PDA-Sr/AMP has a strontium content of 1.89% (w/w) (Figure 2C). On PEEK-PDA-Sr/AMP implants, Sr was homogeneously distributed, based on the EDS mapping results (Figure 2D). Sr and AMP co-modification on the PEEK implant was further confirmed by XPS analysis of the surface elemental compositions (Figure 2E). On the bare PEEK surface, the only signal peaks detected were those of carbon and oxygen, while Sr 3d signal peaks (134.25 eV) were found in the groups of PEEK-PDA-Sr and PEEK-PDA-Sr/AMP. Besides, the groups of PEEK-PDA, PEEK-PDA-Sr and PEEK-PDA-Sr/AMP all found the 1 s signal of nitrogen (N1s, 399.87 eV). A piecemeal increase in N1s can be seen when the PDA treatment PEEK surface was further modified with antimicrobial peptide PMAP-36 (PEEK-PDA-Sr/AMP groups). According to the calculation, 2.24% N (w/w) from PEEK-PDA group and 2.49% N (w/w) from PEEK-PDA-Sr group, in contrast, PEEK-PDA-Sr/AMP group has more than 1.7-fold to reach 4.35% N (w/w). Sr and nitrogen atom percentages on the Sr/AMP surface were found to be 2.97% and 4.35%, respectively, by quantitative analysis, demonstrating the effectiveness of co-modification of Sr ions with antimicrobial peptides (Figure 3C). Besides, the hydrophilic/hydrophobic properties of the different PEEK samples were presented by the water contact angle. From Figures 3A,B, the bare PEEK’s contact angle was 89.9 ± 2.62, decreasing to 60.7 ± 6.95 of PEEK-PDA, 59.1 ± 5.51 of PEEK-PDA-Sr, and 57.9 ± 5.01 of PEEK-PDA-Sr/AMP. Therefore, the PDA decorated PEEK surface demonstrated better wettability compared with bare PEEK surface (*p* < 0.01). What’s more, the hydrophilicity of PEEK implants was not appreciably altered by the alteration of Sr and AMP.

### 3.2 Release behavior of Sr and AMP

20.04 ± 2.12 μg/mL Sr^2+^ was released from PEEK-PDA-Sr/AMP at the initial 24 h (Figure 3E), and 53.31 ± 1.87 μg/mL Sr^2+^ on 7 days, which implied that Sr^2+^ released in bursts during the first 7 days and the release slowed down in the following few days. After 21 days, Sr^2+^ can be found on PEEK-PDA-Sr/AMP at 63.79 ± 1.60 μg/mL. Figure 3F shows the release of AMP from PEEK-PDA-Sr/AMP at different time points and the release curve shows similar biphasic release behavior. Within the first 7 days, AMP displayed a burst release, which reached 45.9% ± 1.7% from PEEK-PDA-Sr/AMP. Then the release percentage of AMP showed a much slower and lasting 3 weeks, which reached 55.19% ± 1.82% after 21 days of incubation.

### 3.3 Biocompatibility assessment of PEEK implants

**FIGURE 4:**
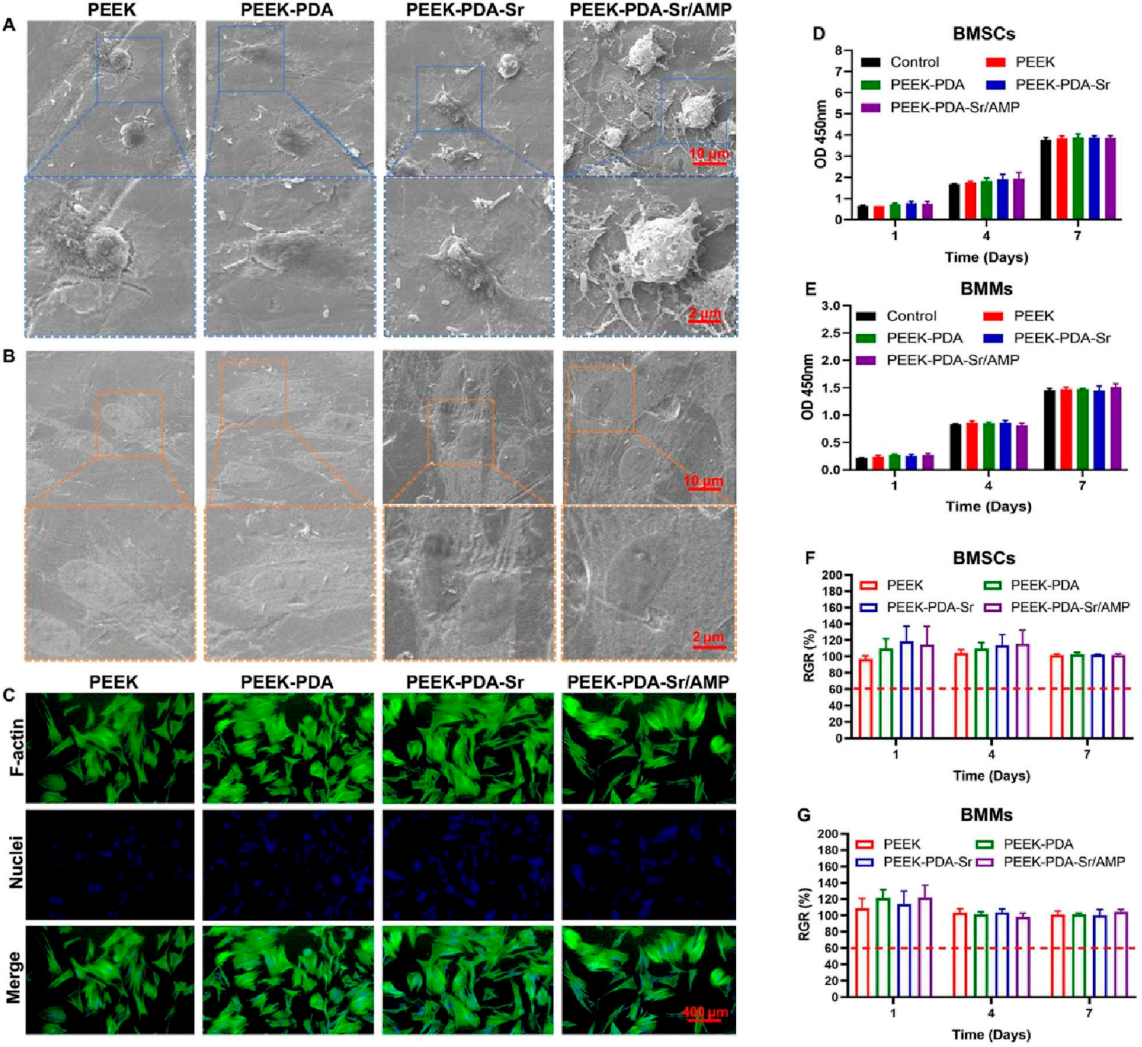
The biocompatibility of Sr^2+^ and AMP co-modified PEEK surfaces. **(A,B)** SEM images of BMSCs and BMMs culturing on different PEEKs for 1 day. Scale bars, 10 μm and 2 µm. **(C)** The cytoskeleton staining (FITC phalloidin/DAPI) of BMSCs on different PEEK surfaces for 1 day. Scale bars, 400 µm. **(D,E)** Cell viability of BMSCs and BMMs on different PEEKs for 1, 4, and 7 days (CCK-8). **(F,G)** Calculated RGR values of BMSCs and BMMs on various PEEK surfaces with different incubation durations.

Cell adhesion and proliferation serve as the fundamental criteria for evaluating a biomaterial’s biocompatibility. Following seeding on PEEK implants, BMSCs and BMMs were analyzed using SEM, fluorescence microscopy, and CCK-8. Following 1 day of culture, the morphology of BMSCs adhered on modified PEEK implants showed well-spread morphology with flat and irregular shapes (Figure 4B) compared to BMSCs on bare PEEK. Moreover, BMMs on modified PEEK materials also presented plentiful filopodia and homogeneous distribution compare with exhibited limited cell spreading bare PEEK (Figure 4A). After 24 h of incubation, the BMSCs showed higher levels of F-actin expression, more extended morphologies, more remarkable intercellular interaction, and more luxurious filopodia on PEEK with surficial treatments than that on bare PEEKs, according to additional cytoskeleton staining (FITC-phalloidin/DAPI) studies (Figure 4C). There were no obvious variations in surface morphology between modified PEEK implants, which matched the SEM findings. The surficial changes on PEEK implants can enhance cell adherence and spreading, as demonstrated by the results of fluorescence microscopy and SEM.

Additionally, the CCK-8 assay of BMMs and BMSCs was used to examine the cytotoxicity of different PEEK implants. From Figures 4D,E, the number of both BMSCs and BMMs presented an obvious increase on modified PEEK implants at days of 1, 4, and 7. These four varieties of PEEK implants did not significantly differ in terms of cell survival at every time point (p > 0.05). Furthermore, on the surface of modified PEEK implants, the corresponding growth rate of these two cell types was higher than 75% at different times (Figures 4F,G). Consequently, the modification of PDA and decoration of Sr/AMP on PEEK materials can increase the proliferation of cells without obvious cytotoxicity.

### 3.4 The effects of PEEK implants on polarization and inflammatory response in BMMs

**FIGURE 5:**
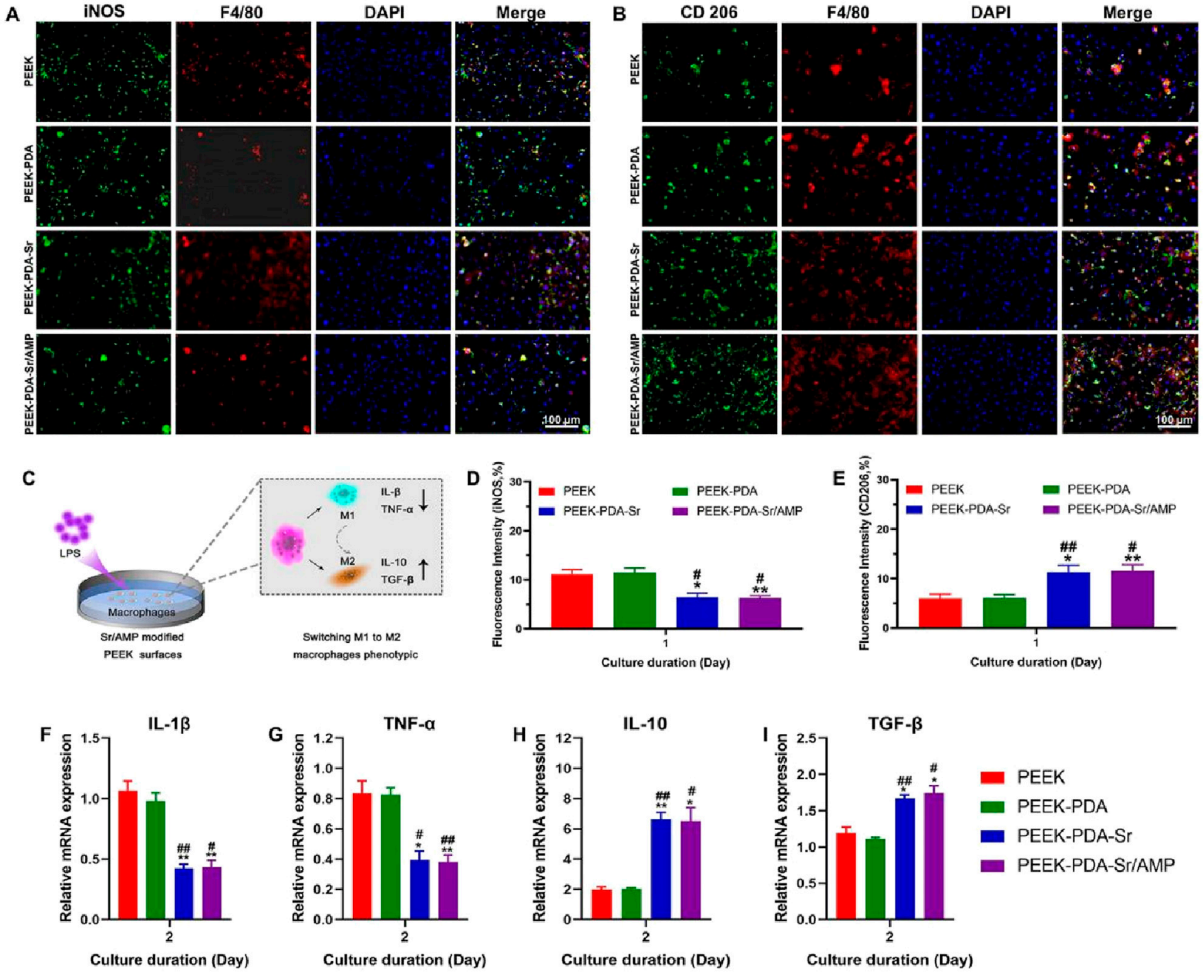
Sr^2+^ and AMP co-modified PEEK surfaces regulate macrophage polarization *in vitro*. **(A,B)** Representative immunofluorescence images of M1 BMMs (iNOS positive, double-stained with DAPI) and M2 BMMs (CD206 positive, double-stained with DAPI) cultured on different samples. Scale bars, 100 µm. **(C)** The illustration of experimental design. **(D**,**E)** The semi-quantified fluorescence intensity of the positive M1 and M2 macrophages. **(F–I)** RT-PCR analysis of macrophage polarization and inflammation-related gene expression after 2 days of culture. Data are presented as mean ± SD (*n* = 3). **p < 0.01 and *p < 0.05 vs. PEEK; ^##^ *p* < 0.01 and ^#^ *p* < 0.05 vs. PEEK-PDA.

In order to examine the impact of our approach on immune microenvironment modulation, we examined macrophage polarization on the surfaces of different PEEK implants. From Figures 5A,B, the findings of immunofluorescence staining exhibited that higher levels of iNOS and lower levels of CD206 were seen from BMMs cultured on non-Sr modified PEEK materials. On the other hand, the Sr-modified PEEK materials showed incremental CD206 and reductive iNOS production, indicating that Sr ions modulate the M1 to M2 macrophage phenotype. These findings were also supported by semi-quantification of fluorescence intensity (Figures 5D,E). The Sr ions-modified PEEK groups had a larger percentage of M2 macrophages (CD206), and the fraction of cells expressing iNOS was likewise lower. Meanwhile, there was not a noticeable distinction in the percentage of cells expressing CD206 and iNOS in the non-Sr-modified PEEK groups.

In order to investigate possible mechanisms behind the differentiation of BMMs, RT-PCR was used to quantify the expression of inflammatory genes such TGF-β, IL-10, IL-1β, and TNF-α. The non-Sr-modified PEEK groups did not present a statistically significant change in the levels of gene expression, as shown in Figures 5F–I. Without observable differences, PEEK groups treated with Sr ions were able to upregulate the expression levels of M2 macrophage-related genes, including TGF-β and IL-10, and downregulate the expression levels of M1 macrophage-related genes, such as TNF-α and IL-1β. These findings also demonstrate the impact of Sr-modified PEEK groups on macrophage phenotypic switching to M2 and improving bone healing potentials at the interface between the implant and bone.

### 3.5 Osteogenesis abilities of PEEK implants in BMSCs

**FIGURE 6:**
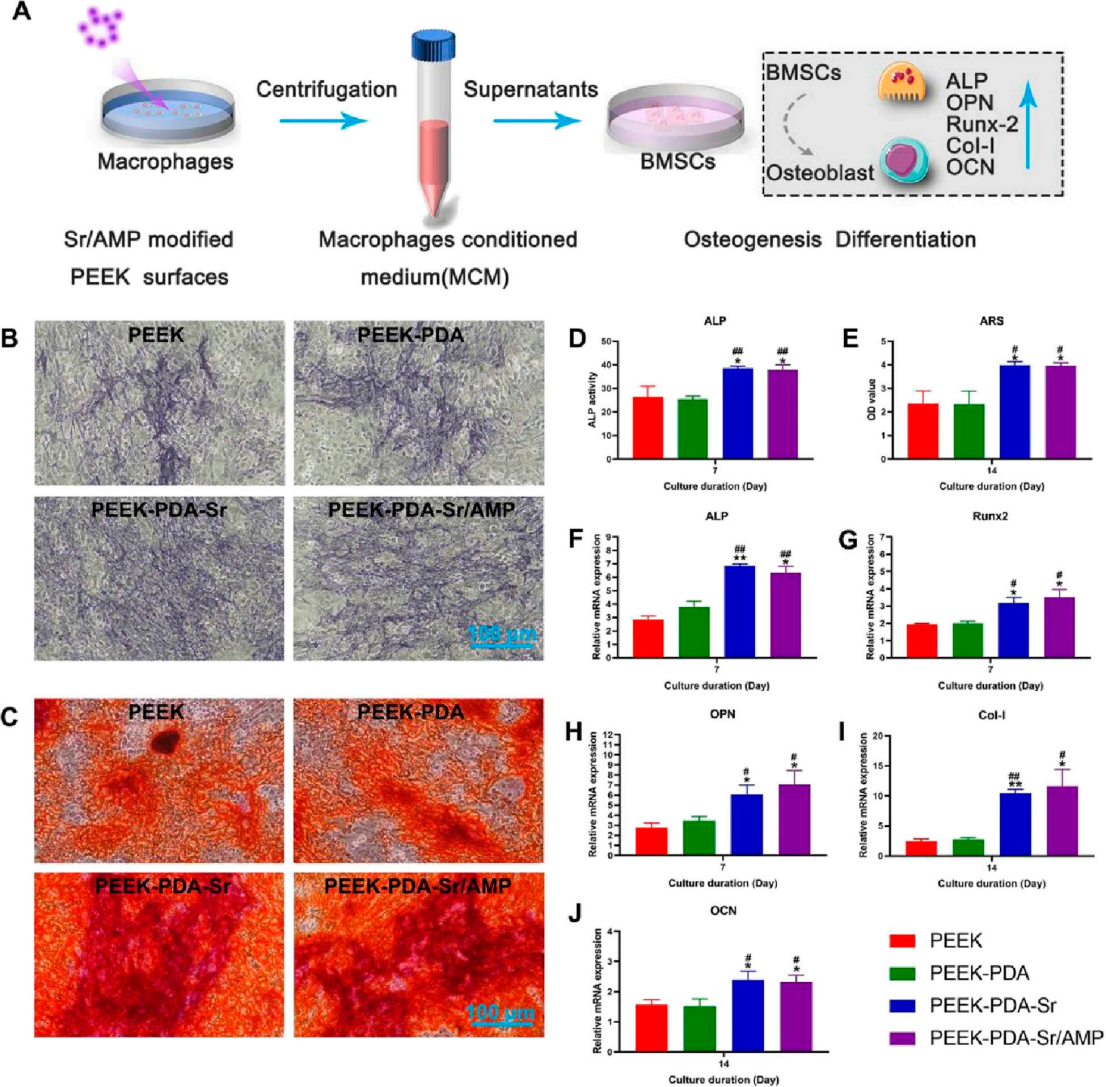
Sr^2+^ and AMP co-modified PEEK surfaces enhance osteogenic differentiation *in vitro*. **(A)** The illustration of experimental design. **(B)** ALP staining and **(C)** ARS staining of BMSCs cultured in osteogenic medium supplemented with MCM. Scale bars, 100 µm. **(D**,**E)** Quantitative analysis of ALP and ARS staining (*n* = 3). **(F–J)** Gene expressions of osteogenesis-related proteins including ALP; Runx2; OPN; Col-I and OCN of BMSCs in different groups for 7 and 14 days. **p < 0.01 and *p < 0.05 vs. PEEK; ^##^ *p* < 0.01 and ^#^ *p* < 0.05 vs. PEEK-PDA.

BMSCs were grown for seven and 14 days in the macrophage conditioned media to see if controlling the polarization of macrophages may affect osteogenesis. The osteogenesis of BMSCs was assessed by ALP and ARS staining (Figures 6B,C). Following a 7-day incubation period, PEEK implant groups modified with Sr ions showed higher ALP stain intensity than PEEK implant groups that were not changed with Sr ions. Additionally, ECM mineralization of BMSCs can be stained with ARS staining following 14 days co-culture. When compared to PEEK implants without ions modification, the Sr ions-treated PEEK materials showed more mineral nodules. The results of ALP and ARD staining was further validated by the quantitative measurement (Figures 6D,E).

Subsequently, we assessed the osteogenic differentiation ability of BMSCs on various PEEK implants by measuring the mRNA expression of genes linked to osteogenesis (Figures 6F–J). In the early stage, OPN, Runx2, and ALP gene expression increased for BMSCs in the PEEK-PDA-Sr and PEEK-PDA-Sr/AMP conditioned media after 7 days of culturing. In the later stage, after 14 days of culturing, Col-I and OCN gene expression levels rose in BMSCs grown in PEEK-PDA-Sr and PEEK-PDA-Sr/AMP conditioned media. In addition, no degree of gene expression can be increased in BMSCs by PEEK or PEEK-PDA conditioned media. These findings therefore indicated that the Sr-modified PEEK implants induced macrophage immunomodulatory action, which enhanced osteogenic bioactivity in BMSCs.

### 3.6 Anti-infection evaluation of various PEEK implants

**FIGURE 7:**
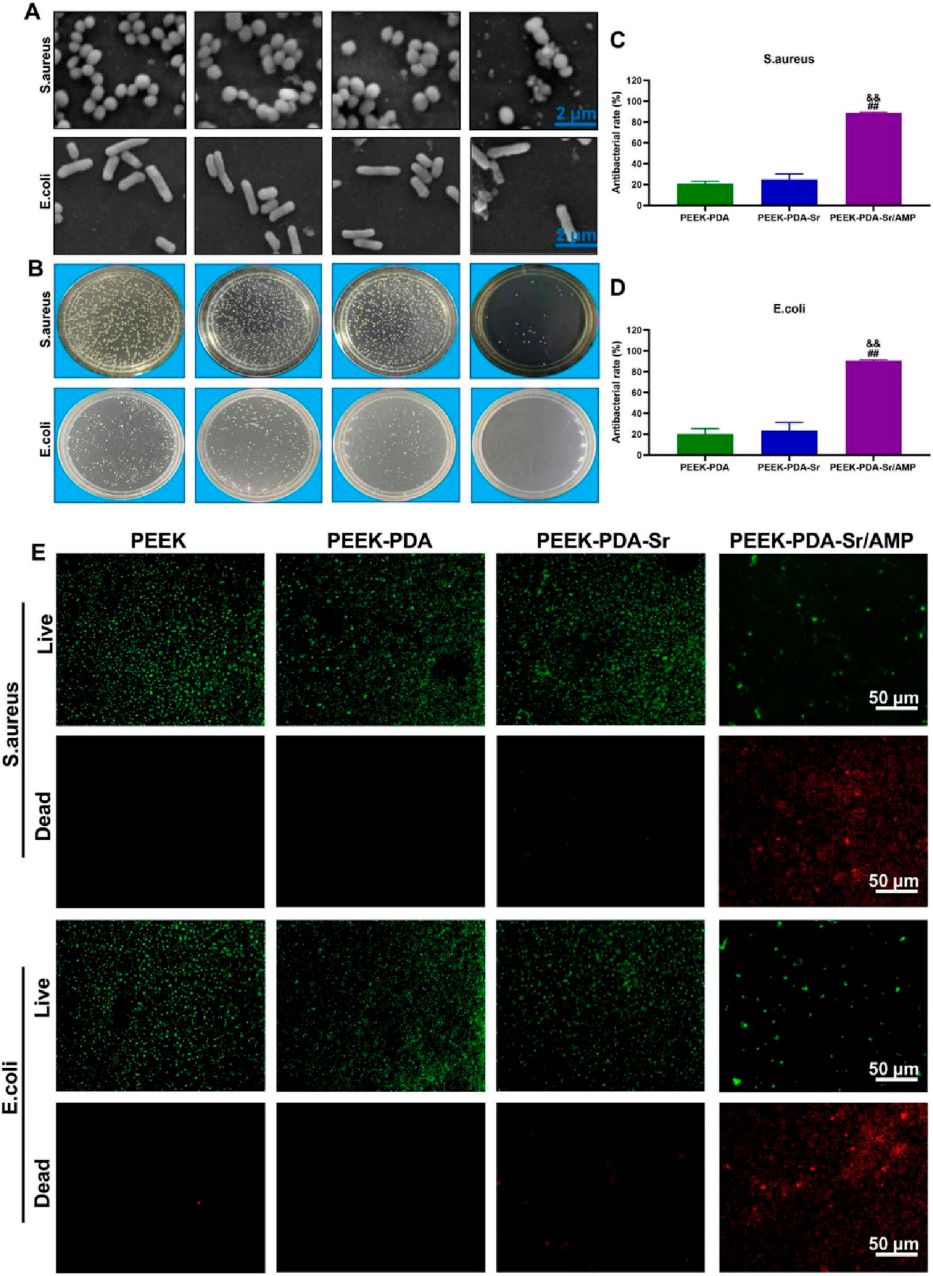
*In vitro* antibacterial ability of different PEEKs. **(A)** SEM Images of *S. aureus* and *E. coli* on the surface of different PEEKs. Scale bars, 2 µm. **(B)** Representative images of bacteria colonies (*S. aureus* and *E. coli*) adhered to different PEEKs after 24 h of culture. **(C,D)** Calculated antibacterial rates of different PEEKs against *S. aureus* and *E. coli* by the plate-counting method (*n* = 3). **(E)** Live/Dead staining of *S. aureus* and *E. coli* on PEEK, PEEK-PDA, PEEK-PDA-Sr and PEEK-PDA-Sr/AMP, the green fluorescence referred to live bacteria and red referred to dead bacteria. Scale bars, 50 μm ^##^ *p* < 0.01 vs. PEEK-PDA; ^&&^ *p* < 0.01 vs. PEEK-PDA-Sr.

We first used SEM to examine the distribution and morphology of bacteria on the samples to evaluate the antibacterial efficacy of various PEEK implants (Figure 7A). In the non-AMP modified PEEK groups, *S. aureus* presented a complete smooth spherical shape. In contrast, the fragmentation and irregular flocculation structure of *S. aureus* were observed in Sr/AMP co-modified PEEK groups. In addition, the morphology of *E*. *coli* maintains complete and intact, while the appearance collapse of *E*. *coli* with fragments of dead *E*. *coli* was observed on surface of Sr/AMP co-modified PEEK groups. The same pattern was also seen in the findings of the live/dead staining of bacteria, where green indicated living bacteria and red indicated dead bacteria as well as bacterial cells with broken membranes. As shown in Figure 7E, lots of *E*. *coli* and *S. aureus* can be seen on the non-AMP-modified PEEK materials (green dots). In contrary, many red dots were found on the Sr/AMP co-modified PEEK groups, which reveals that most bacteria of both *E*. *coli* and *S. aureus* are dead. Compared to the other three groups, the Sr/AMP co-modified PEEK group exhibited the fewest bacterial colonies and the most prominent antibacterial ability (Figure 7B). The antibacterial rates of several PEEK implants were clearly shown in Figures 7C,D. PEEK-PDA-Sr/AMP had an average antibacterial rate of 88.6% on *S. aureus*, which was greater than PEEK-PDA’s (24.77%) and PEEK’s (20.75%) antibacterial rates (p < 0.01). Furthermore, PEEK-PDA-Sr/AMP demonstrated an average antibacterial rate of 90.48% against *E. coli*, which was greater than that of PEEK-PDA (23.58%) and PEEK-PDA-Sr (20.21%) (p < 0.01). The above *in vitro* antibacterial experiments implied that the addition of antimicrobial peptides significantly promoted the antibacterial ability of PEEK and effectively inhibited Gram-negative *E*. *coli* and Gram-positive *S. aureus*.

### 3.7 Micro-CT analysis

**FIGURE 8:**
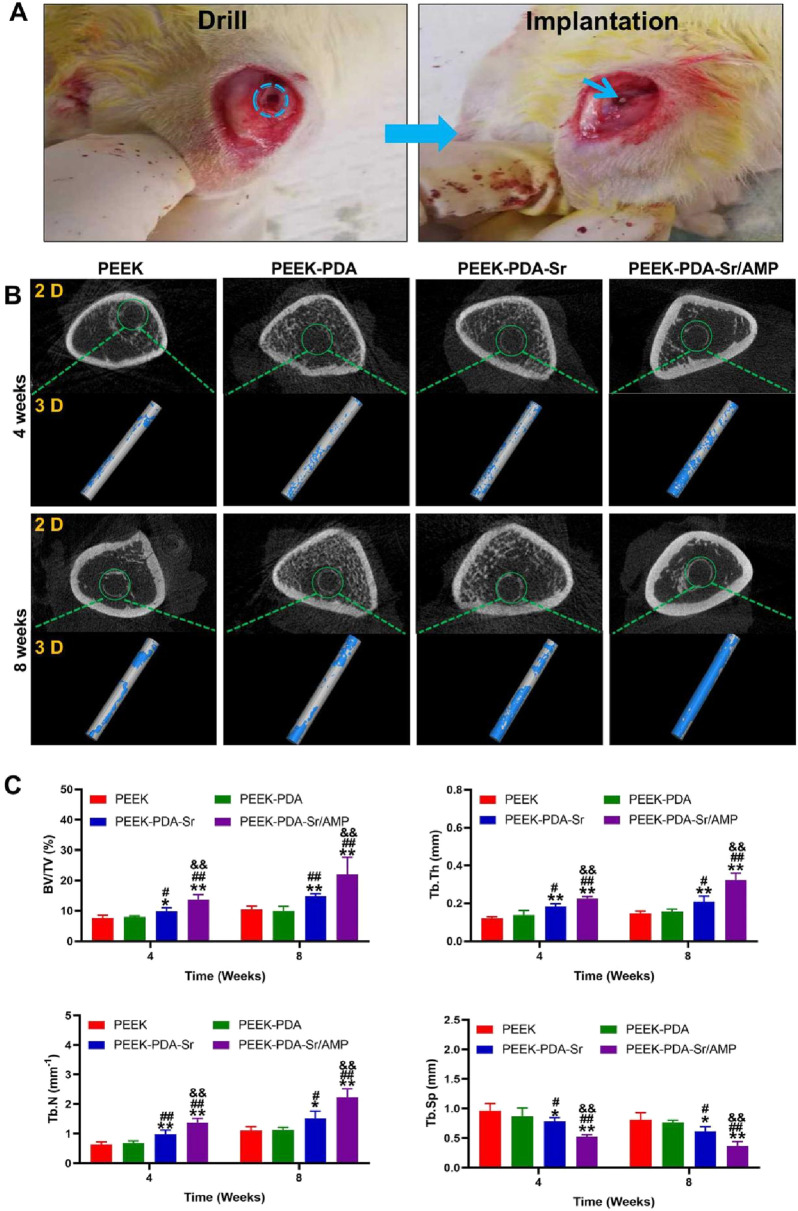
Multifunctional PEEK implants promoted osseointegration in the presence of S. aureus *in vivo.* **(A)** Establishment of femur implant-associated infection model in SD rats. **(B)** Representative 2D and 3D reconstruction images of the femur treated with different PEEK implants at 4 and 8 weeks after surgery (the grey color represented the implant and the blue color represented the new bone tissue). **(C)** Quantitative analysis of the peri-implant bone generation according to the BV/TV, Tb. N, Tb.Th and Tb. Sp on the different groups (*n* = 4). **p < 0.01 and *p < 0.05 vs. PEEK; ^##^ *p* < 0.01 and ^#^ *p* < 0.05 vs. PEEK-PDA; ^&&^ *p* < 0.01 vs. PEEK-PDA-Sr.

Micro-CT was utilized to analyze bone repair around the implants in each group under bacterial infection conditions. Figure 8B shows two columns of representative 2D and 3D photos of each group at various time points. At 4 weeks after surgery, PEEK, PEEK-PDA, and PEEK-PDA-Sr groups showed decreased new bone formation and considerable structural bone degradation around the implants, suggesting that these groups were unable to regulate the infection process by preventing bacterial growth, which hindered the bone formation process. In contrary, PEEK-PDA-Sr/AMP group did not observe significant bone tissue destruction around the implant, while inducing more bone formation around the implant, indicating that its good antibacterial can successfully prevent infection of bone tissue and encourage integration of bone tissue. The results of 8 weeks after surgery showed the same trend as those of 4 weeks after surgery. In addition, the results of relevant bone formation parameters were obtained based on the analysis of Micro-CT data at 4 and 8 weeks postoperatively (Figure 8C). Compare with other three groups, the higher values of Tb. Th, BV/TV, Tb. N and the lower level of Tb. Sp were found in PEEK-PDA-Sr/AMP group, which further proves that Sr/AMP co-modified PEEK implant can effectively preventing bone metabolism disorders caused by osteomyelitis and promoting bone regeneration.

### 3.8 The effects of PEEK implants on polarization response *in vivo*

**FIGURE 9:**
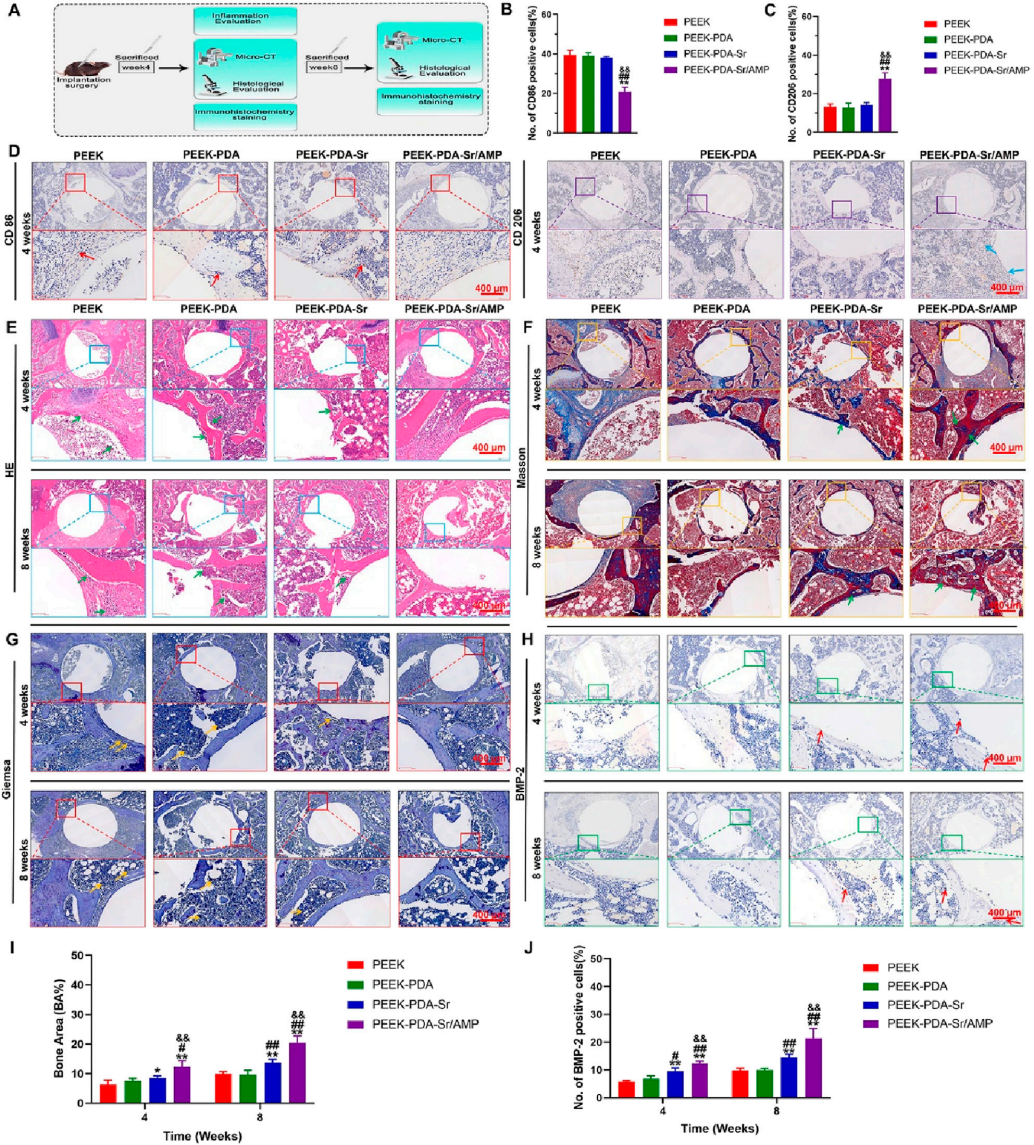
Histological analysis and immunohistochemical staining in the bone tissue treated with different PEEK implants. **(A)** Schematic illustration of surgery and treatment process *in vivo*. **(B,C)** Semi-quantitative analysis on the number of CD86 and CD206 positive cells (*n* = 4). **(D)** Representative images of immunohistochemical staining (CD86 and CD206) in the peri-implant bone tissue after implants implantation for 4 weeks. Scale bars, 400 µm. **(E)** HE staining images at 4 and 8 weeks after surgery, the green arrows mark the neutrophil infiltration in the tissue around samples. **(F)** Masson’s trichrome staining images at 4 and 8 weeks after surgery, the green arrows mark the new bone tissues around the implants. **(G)** Giemsa staining images at 4 and 8 weeks after surgery, the yellow arrows mark the bacterial residues in the tissue around samples. **(H)** Immunohistochemical images of BMP-2 images at 4 and 8 weeks after surgery, the red arrows mark the BMP-2 positive cells around the implants. The local magnifying images are exhibited in the lower lane. Scale bars, 400 µm. **(I**,**J)** Semiquantitative analysis of the bone area and BMP-2 positive cells around different PEEK implants at 4 and 8 weeks after surgery (*n* = 4). **p < 0.01 and *p < 0.05 vs. PEEK; ^##^ *p* < 0.01 and ^#^ *p* < 0.05 vs. PEEK-PDA; ^&&^ *p* < 0.01 vs. PEEK-PDA-Sr.

Figure 9D shows the inflammatory reaction process of PEEK implants in different groups at 4 weeks after surgery. Using immunohistochemical labelling, the expression levels of the M1 phenotype macrophage marker CD86 and the M2 phenotype macrophage marker CD206 in rat femur tissues were examined. In brief, compared with PEEK, PEEK-PDA and PEEK-PDA-Sr groups, PEEK-PDA-Sr/AMP exhibits higher CD206 expression and lower CD86 expression at 4 weeks after the surgery, indicating that co-modified PEEK with Sr/AMP could cause an M2 macrophage transition and create an anti-inflammatory milieu under conditions of bacterial infection (Figures 9B,C).

### 3.9 Pathological histology and immunohistochemistry

Hematoxylin-Eosin (H&E), Giemsa staining, Masson staining and BMP-2 immunohistochemistry staining were utilized to evaluate the osseointegration of PEEK materials in infected bone defects of rat femur. As shown in Figure 9E, HE staining displayed that the bacterial infection damaged bone tissue with a large number of neutrophils in the bone marrow cavity around PEEK, PEEK-PDA and PEEK-PDA-Sr implants following 4 weeks of surgery. Following 8 weeks of implantation, the surrounding medullary tissue of the non-AMP modified PEEK implant showed a significant increase in chronic inflammatory cells, which means there has been implant-related osteomyelitis. At the same time, Giemsa staining showed bone tissue surrounding PEEK, PEEK-PDA and PEEK-PDA-Sr groups had many bacterial residues. In contrast, no evident inflammatory reactions or bacterial colonies were observed around the bone tissue of the PEEK-PDA-Sr/AMP, indicating that the incorporation of AMP has produced good antibacterial effect (Figure 9G). Base on the Masson trichrome staining results (Figure 9F, limited new bone tissues with fibrous tissues can be observed around the non-AMP-modified implants interface following 4 and 8 weeks of surgery. By comparison, many new bone tissues accompanied by decreased fibrous tissues can be observed around the Sr-AMP co-modified implants interface. The results of the Masson trichrome staining agreed with the Micro-CT findings. Furthermore, compared to the other three groups, a greater number of BMP-2 positive cells were seen surrounding the Ti-PDA-Sr/AMP implants after 4 and 8 weeks of implantation (Figures 9H,J). In the case of bacterial infection, Ti-PDA-Sr/AMP implants can successfully increase osseointegration *in vivo*, as further indicated by the immunohistochemical staining results that matched the quantitative results of the bone area (Figure 9I).

## 4 Discussion

In orthopedic surgery, biomaterials are frequently utilized as bone implant materials to treat patients with injuries, tumors, infections, osteonecrosis, and other pathological conditions. Good osseous integration, or the capacity of implant materials to adhere to the surrounding new bone tissue without producing fibrous tissue, is the most important component for the long-term durability of orthopedic implants *in vivo* (Torstrick et al., 2018; Chen et al., 2023). Due to PEEK is a bioinert material, fiber cysts formed upon implantation prevent the implant from making direct touch with the surrounding bone tissue, which eventually causes the implant to loosen or perhaps collapse (Chen et al., 2023; Yuan et al., 2018). Postoperative implant-associated infections (IAI) are another serious complication which make bone integration process more complex, treatment IAI often includes antibiotic system administration, debridement surgery, and implant removal, which has negative effects on the operative effect and patient’s quality of life (Heras et al., 2020). Based on modelling data, it is predicted that by 2030, the prevalence of prosthetic infections resulting from hip and knee replacements will surpass 6%. This growth can be attributed to various variables, including an ageing population, growing surgical demand, and the obesity pandemic (Heras et al., 2020; Peel, 2019). Prior research mostly concentrated on enhancing PEEK’s osteogenic qualities and antibacterial potential using a variety of modification strategies. However, the field of osteoimmunology has garnered a lot of interest because of the inconsistent results of direct osteogenic biomaterials *in vitro* and *in vivo* (Liu et al., 2018). Further research suggests that the immune response triggered by biomaterials is the cause of this phenomenon, which has been shown that the bone immune cells can affect bone remodeling and absorption since immune cells in bone and the skeletal system share lot of receptors, cytokines, and signaling molecules (Yu et al., 2022; Zhang et al., 2023; Liu et al., 2018). Research has confirmed that excessive immune and inflammatory responses after implantation surgery can result in the growth of fibrous tissue and the creation of fibrous capsules, which in turn cause the PEEK and bone tissue to become loosely connected and cause implantation failure (Zhang et al., 2023). These results imply that to create a favorable immune microenvironment and achieve satisfactory osseointegration results, cutting-edge methods for bone regeneration materials should concentrate on the development of immunomodulatory materials in addition to osteogenesis and antimicrobial resistance.

When choosing surface modification techniques for PEEK implants, simple yet efficient surface modification techniques documented in the literature are favored to minimize complexity and guarantee repeatability, dependability, and safety, making them more promising for clinical translation (Zheng et al., 2022; Zhang et al., 2023). PDA coatings have been employed extensively recently for implant material surface biofunctionalization because of their high adherence, easy fabrication, and excellent biocompatibility (Jia et al., 2019; Cheng et al., 2019). For most of the bone implants, PDA often acts as a link and reaction platform, which can chelate metal ions or graft biomolecular via Schiff base reaction and Michael addition for biomedical applications (Wang T. et al., 2022; Wang L. et al., 2023; Zhang X. et al., 2022; Wang et al., 2024). Here, we create co-modified bone PEEK implants with immunomodulatory active ions (Sr^2+^) and antibacterial active molecules (AMP) via mussel-inspired PDA surface modification. PDA-coated PEEK implants have a hydrophilic surface in contrast to bare PEEK implants, although their surface shape does not alter much. Furthermore, in comparison to implants without surface treatment, the PDA, Sr, and AMP deposits result in a rougher surface on the PEEK implants. Other studies corroborated this as well that PDA coating, active metal ions, and/or peptide immobilization could promote the surface hydrophilicity and toughness of bone implants, which induce cell adhesion and growth (Wang T. et al., 2022; Jia et al., 2019; Yang X. et al., 2024). The EDS and XPS results demonstrated that Sr ions and AMP were successfully deposited on the PEEK-PDA-Sr/AMP implants. Furthermore, the release processes of Sr^2+^ and AMP in PEEK-PDA-Sr/AMP materials were evaluated by ICP-AES. In PEEK-PDA-Sr/AMP implants, Sr^2+^ and AMP were released for 7 days, and then consistently for up to 21 days. This is because the oxygen atom in the phenolic hydroxyl group of PDA typically coordinates with Sr^2+^, forming metal-oxygen bonds. The amino group (-NH2) can also form coordination bonds with Sr^2+^ through its lone pair of electrons. Additionally, PDA interacts with AMP through hydrogen bonding and electrostatic interactions. As a result, the loading and sustained release of Sr and AMP are achieved. To put it succinctly, all these findings suggested that the PEEK-based surfaces could achieve bioactivity for a fair amount of time and were effectively co-modified with Sr/AMP.

The biocompatibility of the implant is significantly impacted by the way cells interact with the surface of the biomaterial, and optimal host-implant response depends upon the cell’s ability to adhere to the implant surface (Wei et al., 2023). Based on SEM images, BMSCs and BMMs showed superior morphology and dispersion on the PEEK materials with a PDA coating than bare PEEK implants following a 24-h co-culturing period. Further studies with cytoskeleton staining revealed that BMSCs were a shape of spindle on the bare PEEK surface with a limited expression of F-actin after 24 h. In contrast, on the surface of PDA-treated PEEK implants, BMSCs were polygonal and filamentous, widely distributed, and expressed high levels of F-actin, suggesting improved cell adherence on these samples. Meanwhile, there were no discernible variations in the conditions of cell adhesion and distribution on PDA-modified PEEK implants. The above findings matched previous research, which demonstrating that PDA coatings can increase the hydrophilicity and surface roughness of implants, promote cell adherence and distribution (Wang L. et al., 2023; Jia et al., 2019; Yang X. et al., 2024). Hee K et al. reported that biosafety of PDA surface modification, which does not hinder the proliferation rate or cell activity types of kinds of mammalian cells, including osteoblasts, fibroblasts, endothelial cells, and neurons (Wei et al., 2023). Using CCK-8, the cytotoxicity of several PEEK implant groups was examined, and RGR values were determined. Following 1, 4, and 7 days of co-culture, there was no discernible variation in the OD value across all PEEK implants. Furthermore, all groups’ RGRs were greater than 75%, indicating that all PEEK implants are biocompatible. According to previous research, 0.32–78.34 μg/mL of Sr ions are safe range for BMSCs and BMMs following 7 days of incubation (Wang L. et al., 2023; Xing et al., 2018). Taken together, the findings suggested that the Sr^2+^ and AMP co-modified PEEK surfaces via PDA surface modification do not hinder the cell proliferation of both macrophages and BMSCs. A good milieu for bone tissue regeneration would be built on the immune cells’ and BMSCs’ enhanced adhesion, spreading, and decreased cytotoxicity (Wang T. et al., 2022).

The moment biomaterials are inserted into the body, the host’s local immunological response is triggered. This includes the production of blood clots, the absorption of proteins that create temporary matrices, and an acute inflammatory response. Activated cytokines cause monocytes to congregate around implants during the acute inflammatory phase and undergo differentiation into macrophages, which are crucial for tissue regeneration and the creation of new bone (Zhang et al., 2023; Peng et al., 2022). When it comes to bone implants, excessive pro-inflammatory M1 subtypes result in fibrous capsule development and bone resorption, which cause the implant and bone to become loosely connected, eventually causing implantation failure (Wang L. et al., 2023). Related studies have shown that PEEK can be surface modification via techniques such as surface chemical modification, drug delivery systems, and surface porous treatment to endow PEEK implants with anti-inflammatory and immunomodulatory functions to differentiate pro-inflammatory M1 subtype into anti-inflammatory M2 subtype and promote tissue repair is an effective strategy (Zhang et al., 2023; Yang X. et al., 2024). The immunofluorescence results of this investigation demonstrated that the Sr-modified PEEK groups promoted the expression of CD206 (a marker of the M2 phenotype) than the non-Sr-modified PEEK groups. According to these findings, exogenous materials such as PEEK and PEEK-PDA groups would cause macrophages to change from M0 to M1, inducing several harmful host immunological reactions. On the other hand, the addition of Sr by PDA was a successful surface modification method that changed the intracellular milieu by triggering a signaling cascade and modulating the surface roughness of PEEK, giving PEEK osteoimmunomodulatory characteristics. Furthermore, semi-quantitative results displayed that Sr ions-modified PEEK material can differentiate BMMs into M2 subtypes with fewer M1 macrophages. Recent studies have demonstrated that the NF-kB signaling pathway is a considerable regulator of the immune response, and the receptor activator of NF-kB ligands (RANKL) is thought to be a crucial component that links the skeletal system and the immune system (Peng et al., 2022; Yang S.-Y. et al., 2024). Sr^2+^ can suppress the NF-κB signaling pathway and repolarize M1 macrophages into M2 macrophages to reduce the inflammatory response which helps with tissue repair (Yang X. et al., 2024; Zeng et al., 2020).

To investigate the immunomodulatory mechanism of PEEK implants treated with Sr ions, RT-PCR was further utilized to analyze the BMMs seed on various PEEK implants. PEEK implants with Sr ion can suppress the gene expression of pro-inflammatory factors including IL-1β and TNF-α, and promote the gene expression of anti-inflammatory factors including IL-10 and TGF-β. This is in line with earlier studies because, when macrophages polarize to the M1 subtype, NF-κB is phosphorylated and transported into nucleus, forming pro-inflammatory cytokines like IL-6, TNF-α, and IL-1β that are detrimental to tissue healing (Yang X. et al., 2024; Chen et al., 2020). Based on the above results, we can conclude that Sr ions-modified PEEK implants can create an advantageous immune microenvironment by modulating the polarization of macrophages towards M2 subtype, which benefits for subsequent bone repair.

The concept behind “osteoimmunomodulation” is to control macrophages to create an environment that is favourable for osteogenesis, thereby improving bone integration of implantable biomaterials (Zhang et al., 2023; Wang T. et al., 2022). Generally speaking, acute and uncontrollable inflammation reaction can damage osteogenic differentiation and bone regeneration, while a mild immune response to the biomaterial was favorable for promoting bone integration of the implant (Wang F. et al., 2023). Thus, we cultivated BMSCs in macrophage-conditioned media to assess the osteogenic differentiation capacity of the environment generated by Sr-modified PEEK. In current study, a Sr-modified PEEK implants was developed, which can promote the gene expression of ALP, Runx2, COL-I, OCN, and OPN in BMSCs. Runx2 is a particular transcription factor that regulates the expression of matrix proteins in osteoblasts, which is essential for osteogenic differentiation and bone production (Wei et al., 2023). Osteoblasts produce COL-I, which is a crucial component of bone matrix and is involved in adhesion, differentiation, and bone matrix production (Bou Assaf et al., 2019). The upregulation of Runx2 and COL-I gene expression indicates that Sr-modified PEEK implants can simultaneously promote early and late osteogenesis. In addition, the mineralization ability of BMSCs on various PEEK implants were determined by ALP and ARS. BMSCs co-cultured with PEEK implants modified with Sr^2+^ exhibited more extracellular matrix mineralization than PEEK implants without Sr modification, which were confirmed by ALP, Alizarin red staining and quantitative analysis. Other research has yielded comparable findings, indicating that strontium functionalized biomaterials have good osteogenic effects (Wang et al., 2025; Mo et al., 2023; Wang L. et al., 2023). The osteogenic effect of Sr ion is concentration-dependent, and the ideal concentration is between 1 and 500 mM to promote osteogenesis *in vitro*; if the Sr concentration exceeds this range, it would hinder its osteogenic potential and cause cytotoxicity (Wang L. et al., 2023; Naruphontjirakul et al., 2022). Sr also can inhibit osteoclast precursor differentiation by downregulating the expression of osteoclast marker genes and decreasing the osteoclast specific protein activity, thus activating the ERK signaling pathway and inhibiting the signaling pathways of p38, JNK, and AKT (Chen F. et al., 2022). In addition, by downregulating the expression of RANK, cth-k, MMP-9, and c-fos, Sr may prevent macrophages from differentiating into osteoclasts, which will decrease the quantity of osteoclasts and bone resorption (Zhu et al., 2016). According to the above findings, it can be indicated that Sr-modified PEEK implants would offer an ideal osteoimmunomodulatory microenvironment and promote immuno-enhanced osteogenesis.

In orthopedic surgery, implants are highly susceptible to infection in the body, which is another major reason for implant failure (Zheng et al., 2022; He et al., 2023). Surgical site infections are becoming more commonplace and is estimated at 2.1%–8.5% in implant surgery (Zheng et al., 2022). Research has shown that bacteria are able to adhere to the implant’s surface, proliferate, and form biofilms, which endows the bacteria with antibacterial properties within the body (Geng et al., 2024; Yang et al., 2023). According to the study, the first 6 h following implant surgery are crucial since this is when bacteria tend to stick to the implant’s surface and multiply (Fu et al., 2021; Yang et al., 2023). Therefore, to increase the long-term success of implantation surgery, early bacterial adherence on the surface of PEEK implants must be prevented. At present, the methods to improve the antimicrobial properties of PEEK mainly include sulfonation treatment, antibiotics/metal nanoparticles/antibacterial coating, antimicrobial peptides coating and so on (Zheng et al., 2022). Antimicrobial peptides with broad spectrum and strong antibacterial activity can play an antibacterial role against a variety of microorganisms, including drug-resistant pathogenic bacteria (Wu et al., 2023; Mookherjee et al., 2020). In contrast to physical integration, covalent immobilization is an effective chemical integration method which ensures that antimicrobial peptides stay stable on the surface of the implant material and are not impacted by the surrounding environmental (Wu et al., 2023). Several studies have demonstrated that the abundant functional groups found in PDA coatings, such as the amino and phenolic hydroxyl groups, can act as links to facilitate secondary reactions with other compounds. For example, Michael addition reactions or Schiff base reactions with protein molecules that contain -NH_2_ and -SH groups can be used to adsorb functional protein or peptide molecules onto the surface of biomaterials (Wu et al., 2023; Zhang Y. et al., 2022; Wang B. et al., 2023). Previous publications have found that *S*. *aureus* and *E*. *coli* are the main pathogenic bacteria causing implant infections, we used these two types of pathogens to investigate the antibacterial performance of various PEEK implants (Zheng et al., 2022; Yang et al., 2023). The results of bacterial morphology, live and death staining and spreading plate method all indicate that the Sr/AMP co-modified PEEK implants have remarkable antibacterial properties against *S*. *aureus* and *E*. *coli*. Prior studies have indicated that monomeric forms of the antimicrobial peptide PMAP-36 adopt an amphipathic α-helical conformation and kill bacteria via quickly penetrate both the outer and inner membranes of bacteria by creating toroidal pores or transient channels, which in turn cause bacterial death. At low concentrations, bacteria can be killed, and the minimum inhibitory concentration (MIC) is 1–2 µM for *S*. *aureus* and 0.5–1 µM for *E*. *coli* (Scocchi et al., 2005; Hancock and Chapple, 1999), which is consistent with our experimental results. Above results *in vitro* all indicate that PEEK-PDA-Sr/AMP implants can successfully combine immune regulation, osteogenesis, and antimicrobial properties together with good biocompatibility.

To simulate clinically relevant implant-related infection, we utilized an SD rat femur osteomyelitis model system to further validate its biological function of PEEK-PDA-Sr/AMP implants *in vivo*. It is well known that if bacterial infection and osteomyelitis occur, osseous integration of the implant will be inhibited (Gri and stina, 1987). Micro-CT analysis allows quantitative analysis of bone growth or in-depth observations of infected areas, allowing researchers to accurately compare the development of infection and the process of osseointegration (Wang L. et al., 2023; Lin et al., 2017). The bone tissue surrounding the implant was discontinuous four- and 8-weeks following surgery for the PEEK, PEEK-PDA, and PEEK-PDA-Sr groups, as demonstrated by 2D and 3D reconstruction pictures of the CT scan. This observation implied poor bone integration caused by bacterial infection in these three groups (Gao et al., 2021). On the contrary, PEEK-PDA-Sr/AMP group induced the highest new bone formation around the implant, indicating that Sr-AMP co-modified surface can successfully prevent bone infection and encourage bone integration (Wang et al., 2022b). The quantitative analysis of osteogenic parameters of the PEEK implants including BV/TV and Tb. Sp in different groups also showed the same trend. Extensive literature has confirmed that the increase of M2/M1 macrophages is conducive to promoting the osseous integration of implants (Wang T. et al., 2022; Li et al., 2024). In our case, the expression of CD206 in the PEEK-PDA-Sr/AMP group was highest accompanied by a decrease in CD86 expression at 4 weeks postoperatively, suggesting that the potential of Sr/AMP co-modified PEEK can inhibit inflammatory infection response and enhance interfacial osseointegration. Pathological histological analysis can directly reveal the inflammation, osteogenesis, and bacterial infections *in vivo* (Wang L. et al., 2023; Lin et al., 2017). In current study, H&E, Masson, and Giemsa staining were utilized to evaluate the inflammatory responses, new bone formation and bacterial infections around the implant at different time points, respectively. Similar to the results of *in vitro* studies, *in vivo* models of rat femur implant-associated infections, the PEEK-PDA-Sr/AMP group demonstrated excellent performance in eliminating bacterial infection and promoting bone integration. Previous publications have revealed that PEEK implants with immunomodulatory and antibacterial functions can establish an anti-inflammatory microenvironment, resist pathogen invasion, and increase bone integration under infection conditions (Xu et al., 2019; Su et al., 2023), this is consistent with our findings. As a bone-inducing protein, bone morphogenetic protein 2 (BMP-2) is a potent inducer of bone production and is crucial for the repair of bone tissue (Smith et al., 2023). Sun, et al. reported that successful preparation of 3D bio-printed scaffolds loaded with macrophages and BMSCs, the inflammatory response was inhibited and the secretion of BMP-2 by macrophages was facilitated by inducing the polarization of macrophages to M2 macrophages, thus further accelerating the bone repair of diabetic bone defects (Sun X. et al., 2021), which in line with our results of immunohistochemical staining. Moreover, PDA application *in vivo* is regarded as safe; intravenous infusion of PDA nanoparticles has an LD50 of 400.22–585.19 mg/kg, showing low toxicity (Wang et al., 2022c). In short, the *in vitro* and *in vivo* results confirm that the combination of Sr and AMP can confer multiple biological activities of immunomodulatory, osteogenic, and antibacterial activities on PEEK bone implants, which effectively promote bone integration in the presence of bacteria and may offer a promising approach to the surface bioengineering of inert medical implants, particularly by enabling the rational integration of multiple biofunctions to meet clinical requirements.

Despite the significant progress achieved in this study, several areas require further investigation. For instance, although Sr^2+^ and AMP exhibited sustained release profiles and short-term bioactivity *in vitro*, the long-term structural integrity and functional stability of the coating under physiological conditions—such as mechanical loading, biodegradation, and fluid shear stress—remain to be validated through extended *in vivo* studies and accelerated aging models. Moreover, while the SD rat model is widely accepted for preliminary evaluation of osseointegration and antibacterial performance, it differs substantially from human physiology in terms of bone metabolism, immune complexity, and infection progression. Therefore, we acknowledge that the current findings need to be further verified in large animal models (e.g., rabbits, dogs, or sheep) and in long-term implantation studies to fully assess the feasibility and safety of the proposed strategy.

## 5 Conclusion

To better fulfil the characteristics and requirements of PEEK materials for orthopedic applications, multifunctional PEEK implants with Sr/AMP co-modified were fabricated using mussel adhesion-mediated assembling strategy. PEEK-PDA-Sr/AMP group exhibited excellent biological functions *in vitro*, including promoting cell adhesion and spreading, immune regulation, osteogenic differentiation, and antibacterial effect. *In vivo*, the PEEK-PDA-Sr/AMP implant markedly enhance the ability of interfacial osseointegration of the implant in the presence of bone infection. This surface modification strategy has the advantages of simplicity, high efficiency, and universal application, which offer a surface modification technique with a strong potential for practical conversion to increase the clinical use of PEEK bone implant materials.

## Data Availability

The original contributions presented in the study are included in the article/Supplementary Material, further inquiries can be directed to the corresponding authors.
