# Supplementary material for: Multifunctional PEEK implants via mussel adhesion-mediated assembly for osteoimmune regulation and antibacterial properties
Source: Front Bioeng Biotechnol. 2025 Sep 9;13:1624106. doi: 10.3389/fbioe.2025.1624106 (PMC12454371; doi:10.3389/fbioe.2025.1624106)
Supplement: Supplementary file 1 [file Table1.docx]

**Multifunctional PEEK Implants via Mussel Adhesion-Mediated Assembly for Osteoimmune Regulation and Antibacterial Properties**

[Lei Wang](https://pubs.rsc.org/en/results?searchtext=Author%3ALei%20Wang),^a,1^  [Qiang Wang](https://pubs.rsc.org/en/results?searchtext=Author%3AQiang%20Wang),^a,1^  [Fan Wang](https://pubs.rsc.org/en/results?searchtext=Author%3AQiang%20Wang),^e,1^ Shouliang Xiong,^a^ [Xin Yang](https://pubs.rsc.org/en/results?searchtext=Author%3AXing%20Yang),^a^ [Jie Z](https://pubs.rsc.org/en/results?searchtext=Author%3AXifu%20Shang)hao,^a^ Xiao Lu,^a^ Yinchang Zhang,^a^ Pingbo Chen,^a^ Surong Qian,^b,^* Guohai Lu,^c,^ *[Chengyong Gu](https://pubs.rsc.org/en/results?searchtext=Author%3AXing%20Yang),^d,^*

a Department of Orthopedics, The First Affiliated Hospital of Wannan Medical College, Wuhu 241001, Anhui, China

b Department of Rehabilitation medicine, Suzhou Municipal Hospital, Nanjing Medical University Affiliated Suzhou Hospital, Suzhou 215008, Jiangsu, China

c Orthopedics and Sports Medicine Center, Suzhou Municipal Hospital, Nanjing Medical University Affiliated Suzhou Hospital, Suzhou 215008, Jiangsu, China

d Anesthesiology Department, Suzhou Municipal Hospital, Nanjing Medical University Affiliated Suzhou Hospital, Suzhou 215008, Jiangsu, China

e Department of Orthopaedics, Shanghai Key Laboratory for Prevention and Treatment of Bone and Joint Diseases, Shanghai Institute of Traumatology and Orthopaedics, Ruijin Hospital, Shanghai Jiao Tong University School of Medicine, 197 Ruijin 2nd Road, Shanghai, 200025, PR China

^1^ These authors contributed equally to this work.

* Corresponding authors.

*E-mail addresses:* [LXL65506@163.com](mailto:LXL65506@163.com) (Surong Qian); [xlcz028@sina.com](mailto:gcygcy1979@163.com) (Guohai Lu); [gcygcy1979@163.com](mailto:gcygcy1979@163.com) (Chengyong Gu);

**Table S1**．Primers used in regulating the differentiation of BMMs

| Gene | Forward (5’-3’) | Reverse (5’-3’) |
| --- | --- | --- |
| IL-1β | CACTACAGGCTCCGAGATGAACAAC | TGTCGTTGCTTGGTTCTCCTTGTAC |
| TNF- α | ATGTCTCAGCCTCTTCTCATTC | GCTTGTCACTCGAATTTTGAGA |
| IL-10 | TTCTTTCAAACAAAGGACCAGC | GCAACCCAAGTAACCCTTAAAG |
| TGF-β | CCAGATCCTGTCCAAACTAAGG | CTCTTTAGCATAGTAGTCCGCT |
| GAPDH | AGGAATTGACGGAAGGGC ACC | GTGCAGCCCCGGACATCT AAG |

**Table S2**．Primers used in regulating osteogenic differentiation of BMSCs

| Gene | Forward (5’-3’) | Reverse (5’-3’) |
| --- | --- | --- |
| ALP | TATGTCTGGAACCGCACTGAAC | CGGGACCATTGGGAACTGATAGG |
| Runx2 | ATCCAGCCACCTTCACTTACACC | ACTAGCAAGAAGAAGCCTTTGG |
| OPN | GCGGTTCACTTTGAGGACAC | TATGAGGCGGGGATAGTCTTT |
| ColⅠ | CAGGCTGGTGTGATGGGATT | CCAAGGTCTCCAGGAACACC |
| OCN | AACGGTGGTGCCATAGATGC | AGGACCCTCTCTCTGCTCAC |
| β-actin | CTCATGCCATCCTGCGTCTG | GGCAGTGGCCATCTCTTGCT |
